# Impact of antipsychotics in children and adolescents with autism spectrum disorder: a systematic review and meta-analysis

**DOI:** 10.1186/s12955-021-01669-0

**Published:** 2021-01-25

**Authors:** Gian Loreto D’Alò, Franco De Crescenzo, Laura Amato, Fabio Cruciani, Marina Davoli, Francesca Fulceri, Silvia Minozzi, Zuzana Mitrova, Gian Paolo Morgano, Franco Nardocci, Rosella Saulle, Holger Jens Schünemann, Maria Luisa Scattoni, On Behalf of the ISACA Guideline Working Group, Raffaella Tancredi, Angelo Massagli, Giovanni Valeri, Corrado Cappa, Serafino Buono, Giuseppe Maurizio Arduino, Alessandro Zuddas, Laura Reali, Massimo Molteni, Claudia Felici, Concetta Cordò, Lorella Venturini, Cristina Bellosio, Emanuela Di Tommaso, Sandra Biasci, Clelia M. Duff, Simona Vecchi

**Affiliations:** 1 Department of Epidemiology, Lazio Regional Health Service, Via Cristoforo Colombo, 112, 00154 Rome, Italy; 2 Department of Psychiatry, University of Oxford, Oxford, UK; 3 Pediatric University Hospital-Department (DPUO), Bambino Gesù Children’s Hospital, Rome, Italy; 4 Research Coordination and Support Service, Istituto Superiore Di Sanità, Viale Regina Elena 299, 00161 Rome, Italy; 5 Department of Health Research Methods, Evidence and Impact (Formerly Clinical Epidemiology and Biostatistics), McMaster GRADE Centre, McMaster University, Hamilton, ON Canada; 6 Department of Medicine, McMaster University, Hamilton, ON Canada

**Keywords:** Autism spectrum disorder, Antipsychotics, D2 blockers, Systematic review, Meta-analysis, Children, Adolescents, Guidelines

## Abstract

**Background:**

The net health benefit of using antipsychotics in children and adolescents with ASD is unclear. This review was performed to provide the evidence necessary to inform the Italian national guidelines for the management of ASD.

**Methods:**

We performed a systematic review of randomized controlled trials (RCTs) comparing antipsychotics versus placebo for the treatment of ASD in children and adolescents. For efficacy, acceptability and safety we considered outcomes evaluated by the guideline panel critical and important for decision-making. Continuous outcomes were analyzed by using standardized mean difference (SMD), and dichotomous outcomes by calculating the risk ratio (RR), with their 95% confidence interval (95% CI). Data were analyzed using a random effects model. We used the Cochrane tool to assess risk of bias of included studies. Certainty in the evidence of effects was assessed according to the GRADE approach.

**Results:**

We included 21 RCTs with 1,309 participants, comparing antipsychotics to placebo. Antipsychotics were found effective on “restricted and repetitive interests and behaviors” (SMD − 0.21, 95% CI − 0.35 to − 0.07, moderate certainty), “hyperactivity, inattention, oppositional, disruptive behavior” (SMD − 0.67, 95% CI − 0.92 to − 0.42, moderate certainty), “social communication, social interaction” (SMD − 0.38, 95% CI − 0.59 to − 0.16, moderate certainty), “emotional dysregulation/irritability” (SMD − 0.71, 95% CI − 0.98 to − 0.43, low certainty), “global functioning, global improvement” (SMD − 0.64, 95% CI − 0.96 to − 0.33, low certainty), “obsessions, compulsions” (SMD − 0.30, 95% CI − 0.55 to − 0.06, moderate certainty). Antipsychotics were not effective on “self-harm” (SMD − 0.14, 95% CI − 0.58 to 0.30, very low certainty), “anxiety” (SMD − 0.38, 95% CI − 0.82 to 0.07, very low certainty). Antipsychotics were more acceptable in terms of dropout due to any cause (RR 0.61, 95% CI 0.48 to 0.78, moderate certainty), but were less safe in terms of patients experiencing adverse events (RR 1.19, 95% CI 1.07 to 1.32, moderate certainty), and serious adverse events (RR 1.07, 95% CI 0.48 to 2.43, low certainty).

**Conclusions:**

Our systematic review and meta-analysis found antipsychotics for children and adolescents with ASD more efficacious than placebo in reducing stereotypies, hyperactivity, irritability and obsessions, compulsions, and in increasing social communication and global functioning. Antipsychotics were also found to be more acceptable, but less safe than placebo.

## Introduction

Autism Spectrum Disorder (ASD) is a neurodevelopmental disorder characterized by persistent impairments in reciprocal social communication and social interactions along with the presence of restricted, repetitive patterns of behaviors, interests, or activities [1]. Two recent studies conducted in Italy reported a prevalence of ASD in children (age range 7–9 years old) of 1.14% and 1.3% [2, 3], consistent with its prevalence in the world, which is reported between 1 and 2% [4]. The male: female ratio is about 4: 1 [5], with 48% of children having intellectual disability [5, 6].

No pharmacological treatment has currently shown to be effective for the treatment of the core symptoms of ASD. In general, pharmacological treatments, combined with psychological interventions, are directed at the treatment of associated symptoms (such as irritability) or coexisting psychiatric conditions (e.g. attention deficit disorder, oppositional disorder, schizophrenia spectrum disorders), which are frequent in patients with ASD [7].

Antipsychotics are used to treat associated comorbidities, such as schizophrenia spectrum disorders (SSD) and behavior disorders [8, 9]. A recent meta-analysis estimated a prevalence rate of SSD symptoms in ASD of 9.6% [10], and vice-versa individuals with SSD have significantly more autistic symptoms than healthy controls [11]. An observational study reported a high incidence of multiple treatment failure when children and adolescents with comorbid ASD and SSD are treated with antipsychotics [12].

A systematic review by Pillay et al. [13] suggested that antipsychotics may be useful in improving core symptoms, particularly stereotyped behaviors and narrow interests. The latest, larger clinical studies have been based on second generation antipsychotics (SGA) such as risperidone, aripiprazole and lurasidone, while older studies studied first-generation antipsychotics (FGA). Few studies compared the effect of two or more antipsychotics [13].

This systematic review was performed within the context of the development of evidence-based guidelines for the diagnosis and management of ASD in children and adolescents for the Italian National Institute of Health (in Italian: Istituto Superiore di Sanità—ISS). This systematic review has been specifically conducted by the Evidence Review Team, based on the manual developed and published by the ISS, to support the ISS autism guidelines panel in formulating recommendations [14, 15]. Following a public application process, the ISS established a multidisciplinary panel, including people with ASD and/or their caregivers, that formulated the following question for its first recommendation:

- Should antipsychotics be used for the treatment of children and adolescents with ASD?

## Methods

The key elements of the review protocol including participants, intervention, comparator, outcomes, study design (PICOS) were developed by the guideline panel. Panel members and evidence review team members declared conflict of interests and all the process was recorded and it is available for consultation upon request to the study authors.

### Population

Children and adolescents aged 0–18 years with a primary diagnosis of ASD. A concurrent secondary diagnosis of another medical condition was not considered as an exclusion criterion.

### Intervention

The following list of FGAs and SGAs was selected by panel members to be investigated in the systematic review, with no limitations of dose and administration route: aripiprazole, clozapine, haloperidol, levosulpiride, lurasidone, olanzapine, risperidone, trifluoperazine. We included also studies in which antipsychotics were used as adjunctive treatment (e.g. in addition to behavioral or other pharmacological interventions).

### Comparisons

Placebo or no intervention.

### Outcomes

To measure the desirable and undesirable effects of the treatment, we assessed the following outcomes:

- Restricted and repetitive interests and behavior,
- Hyperactivity, inattention, oppositional, disruptive behavior disorders,
- Self-harm,
- Insomnia,
- Social communication, social interaction,
- Serious adverse events,
- Emotional dysregulation/irritability,
- Anxiety,
- Adverse events,
- Global functioning, global improvement,
- Quality of life,
- Obsessions, compulsions,
- Dropout due to any cause,
- Dropout due to adverse events.

These outcomes were deemed to be highly relevant to children and adolescents with ASD by the guideline panel.

### Subgroup analyses

We performed subgroup analyses dividing the studies between those that reported Aberrant Behavior Checklist (ABC)—Irritability subscale scores ≥ 18 as the participants’ selection criterion, and those that reported different inclusion criteria or no particular criteria in addition to the diagnosis of ASD. The ABC is a scale empirically developed to assess the effectiveness of psychotropic medications by measuring psychiatric and behavioral disturbances exhibited by individuals with intellectual and developmental disabilities through 5 subscales corresponding to as many domains (irritability [range 0–45], lethargy/social withdrawal [range 0–48], stereotypic behavior [range 0–21], hyperactivity/noncompliance [range 0–48], inappropriate speech [range 0–12], with higher scores indicating worse condition) [13, 16]. We assessed credibility of subgroup effects using the criteria proposed by Sun et al. [17], and we considered it in evaluating the certainty in the evidence of effects [18].

### Types of studies included

Randomized controlled trials comparing antipsychotics with placebo or no treatment in the management of ASD were included. Both parallel, crossover and withdrawal design were included. Quasi-randomized trials, such as those allocating by using alternate days of the week, and open label trials were excluded. For trials that had a crossover design only results from the first randomization period were considered, since carry-over effect could not be excluded [19].

### Literature search

We performed a comprehensive computer literature search of the CENTRAL, PubMed/Medline, Embase, PsycINFO, Web Of Science databases and of trial registers to find relevant peer reviewed articles on the effect of antipsychotics for children and adolescents with ASD from the date of database inception until January 2019. The full search strategies used are available in Additional file 1 and Additional file 2. No date limit and no language restrictions were used. Finally, we hand-searched references from relevant systematic reviews and included studies to identify any RCT missed by the search strategy.

### Study selection and data extraction

Two reviewers (FDC, GD) independently evaluated the retrieved studies for inclusion and assessed the methodological quality of included studies. Information extracted included study characteristics (lead author, publication year, journal), participant characteristics (age range, setting, diagnosis), intervention details (dose ranges, mean doses of study drugs) and outcome measures of interest.

### Data analysis

Data were entered and analyzed using STATA 16.1 software. Since different scales were used in the studies, we analyzed data as continuous outcomes using standardized mean difference (SMD) with 95% confidence intervals, utilizing the random effects model, because a certain degree of heterogeneity was expected among trials [20]. In interpreting SMD values, we considered SMD “small” if < 0.40, “moderate” from 0.40 to 0.70, and “large” if > 0.7. We analyzed dichotomous outcomes by calculating the risk ratio (RR) for each trial with the uncertainty in each result being expressed with 95% confidence interval (CI). Heterogeneity between studies has been investigated by the Q-test, by the I-squared statistic (I-squared equal to or more than 50% was considered indicative of heterogeneity), and by visual inspection of the forest plots.

### Dealing with missing data

We managed missing data according to Higgins et al. [19]. If dichotomous outcome data were still missing, they were managed according to the intention-to-treat (ITT) principle, and we assumed that patients who dropped out after randomization had a negative outcome. Missing continuous outcome data were either analyzed using the last observation carried forward to the final assessment (LOCF) or on an endpoint basis, including only participants with a final assessment. When *p* values, t-values, 95% CIs or standard errors were reported in articles, we calculated SDs from their values [20]. If p values, t-values, 95% CIs or standard errors were not reported at the endpoint, SDs were imputed from their baseline values, or, if baseline values were not reported, from the mean value of SDs of individuals randomized to that drug (or to placebo) in the other included studies [21].

### Risk of bias and overall certainty of evidence assessment

Two authors independently (GLD, FDC) assessed the risk of bias in the included studies using the Cochrane risk of bias assessment tool [19].

The main results of the review were presented in’Summary of findings’ (SoF) tables, as recommended by Cochrane [19, 22], using the methodology developed from the Grading of Recommendations Assessment, Development and Evaluation (GRADE) Working Group [23–25]. The confidence in the effect estimates was evaluated in four levels: high, moderate, low, very low. The results were summarized and presented to the panel through the GRADE evidence to decision (EtD) framework [26, 27]. Here we present the results for the following criteria: desirable effects, undesirable effects, and certainty of evidence. The results for the other EtD criteria will be published elsewere [28, 29].

#### Results

### Selected studies

We retrieved from database searching 1987 citations of which 779 were removed, being duplicates. We excluded 1127 records on the basis of titles and abstracts and retrieved 82 documents in full text: 47 studies have been excluded and 35 full-text articles included since satisfied the inclusion criteria. Reasons for the exclusion of the 46 papers: 27 studies whose comparator did not meet inclusion criteria, being augmentation trials without placebo arm [30–51] or trials comparing two pharmacological interventions without placebo arm [52–56]; seven studies were pooled analyses or post-hoc analyses of randomized controlled trials [57–63]; nine studies whose design did not meet the inclusion criteria [64–72]; two studies for which we were not able to retrieve the full-text [73, 74]; one study included only adults [75]. We retrieved 52 records from trial registers, 22 of which evaluated in full text: five trials were excluded since they did not show any result (clinicaltrials.gov identifiers: NCT00147394 [76], NCT00198107 [77], NCT00468130 [78], NCT01171937 [79], NCT00057408 [80]), four trials considered comparators that did not meet inclusion criteria (clinicaltrials.gov identifiers: NCT00080145 [81], NCT00205699 [82], NCT01333072 [83], NCT01844700 [84]), three trial whose design did not meet inclusion criteria (clinicaltrials.gov identifiers: NCT00166595 [85], NCT00691080 [86], NCT00619190 [87]), and two ongoing trials (clinicaltrials.gov identifiers: NCT02574741 [88], NCT03487770 [89]).

**Fig. 1:**
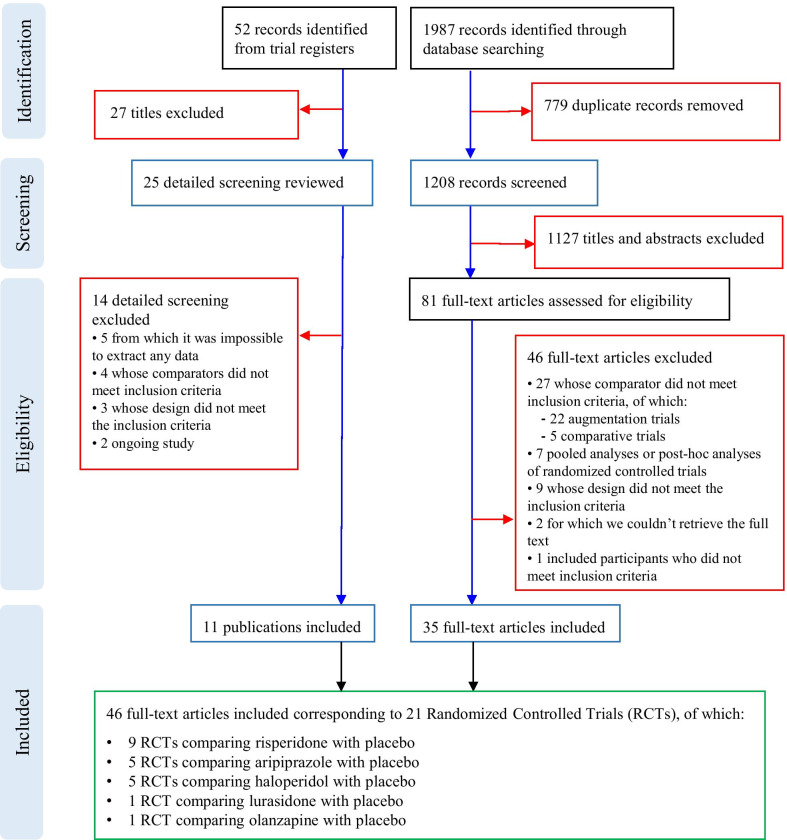
PRISMA Flow chart

Finally, 46 documents, corresponding to 21 RCTs (1309 participants) were included: nine RCTs comparing risperidone with placebo [90–98], five RCTs comparing aripiprazole with placebo [99–103], five RCTs comparing haloperidol with placebo [104–108], one RCT comparing lurasidone with placebo [109], and one RCT comparing olanzapine with placebo [110]. Articles’ selection process is shown in Fig. 1, while full references for included and excluded trials are reported in Additional file 3.

### Included studies characteristics

Six studies (28.6%) included pre-schoolers and school age children, while 15 studies (71.4%) included school-age children and adolescents. The majority were male (83.3%), with a mean age of 8.8 years. The studies included patients diagnosed with autism (71.4%), ASD (23.8%) or PDD-NOS (4.8%), and diagnosis was performed principally through DSM-IV (81.0%) or DSM-III criteria (14.3%). Overall, 318 patients were randomly assigned to aripiprazole, 248 to risperidone, 100 to lurasidone, 67 to haloperidol, 6 to olanzapine, and 545 to placebo. Mean sample size was of 62 (range 11–218). One study recruited patients from Europe, 17 from North America, and three from Asia. Study median duration was eight weeks (Interquartile range: 8–22). Full study characteristics are reported in Table1.

**Table 1:** Characteristics of included Randomized Controlled Trials

Study, Year	Country	Diagnosis	Diagnostic criteria	Severity	Intervention (n)	Control(n)	Study design	Duration of intervention (wks)	Setting	Age mean (SD); range	Female (%)	Mental development	Outcomes	Funding
Anderson, 1984	USA	Autism	DSM-III	Not reported	Haloperidol 0.5 to 4.0 mg/day (20)	Placebo (20)	Crossover	14 (2 weeks SB Placebo baseline, 12 weeks DB intervention: placebo/treatment/placebo or treatment/placebo/treatment)	Inpatient	4.58 (NR); 2.3–6.9	27.5	Not reported	Global changes (CGI-I, CGI-S), Behavior (CPRS), Social Interaction (CPTQ), discrimination learning, adverse events (DOTES), optimal dosages	McNeal Pharmaceuticals; NIMH
Anderson, 1989	USA	Autism	DSM-III	Not reported	Haloperidol 0.25 to 4.0 mg/day (15)	Placebo (30)	Crossover	14 (2 weeks SB Placebo baseline, 12 weeks DB intervention: treatment/placebo/placebo or placebo/treatment/placebo or placebo/placebo/treatment)	Inpatient	4.49 (1.16); 2.0–7.6	22.2	Profound to borderline retardation	Global changes (CGI-I, CGI-S), Behavior (CPRS), Social Interaction (CPTQ), discrimination learning	McNeal Pharmaceuticals; NIMH; March of Dimes Birth Defects Foundation grant 12–108
Campbell, 1978	USA	Autism	Not reported (Kanner and Rutter criteria)	Not reported	Haloperidol 0.5 to 4.0 mg/day, adaptive dose (21)	Placebo (21)	Parallel	12 (2 weeks SB placebo, 3 weeks DB medication, 5 weeks DB medication plus behavioral therapy, 2 weeks SB placebo plus behavioral therapy)	Inpatient	4.5 (NR); 2.6–7.2	20.0	Not reported	McNeal Pharmaceutical; Behavior (CPRS, CBI, NGI), adverse events (DOTES), optimal dosages	NIHM
Cohen, 1980	USA	Autism	DSM-III	Not reported	Haloperidol 0.5 to 4.0 mg/day, adaptive dose (Not reported)	Placebo (Not reported)	Crossover	10 (2 weeks SB placebo, 8 weeks intervention: placebo/Haldoperidol/placebo/Haldoperidol or vice-versa)	Inpatient	4.7 (NR), 2.1–7.0	40.0	Profound to mild retardation	Behavior, adverse events, optimal dosages	McNeal Pharmaceuticals; NIMH
Findling, 2014	USA	Autism	DSM-IV-TR; ADI-R	CGI-S ≥ 4; ABC-Irritability ≥ 18	Aripiprazole 2 to 15 mg, adaptive dose (41)	Placebo (44)	Withdrawal	30 (14 weeks open lable treatment, 16 weeks DB withdrawal)	Specialistic	10.4 (2.8); 6.0–17.0	20.0	MA ≥ 24 months	ABC, Global changes (CGI-I, CGI-S), quality of life (PedsQL), adverse events (AIMS, BARS, SAS)	Bristol-Myers Squibb; Otsuka Pharmaceuticals Co, Ltd
Hellings, 2006	USA	ASD	DSM-IV	ABC-I > 1SD above given norms for age, gender and setting	Risperidone 1.0 mg/day, fixed dose (NR); Risperidone 1.2 to 2.9 mg/day, adaptive dose (NR)	Placebo (NR)	Crossover	22 weeks (4 weeks on average SB placebo, 2 weeks DB drug tapering, 4 weeks DB treatment, 2 weeks DB drug tapering, 4 weeks DB treatment, 2 weeks DB drug tapering, 4 weeks on average SB placebo)	Specialistic	12.0 (2.8); 8.0–15.0	33.3	IQ < 70	ABC, adverse events (DISCUS)	Janssen Pharmaceutica
Hollander, 2006	USA	ASD	DSM-IV; ADI-R; ADOS	CGI-S ≥ 4	Olanzapine 2.5 to 20 mg/day, adaptive dose (6)	Placebo (5)	Parallel	8	Specialistic	9.1 (2.5); 6.0–14.8	18.1	Profound retardation to normal	Global changes (CGI-I), compulsion (CY-BOCS), aggression (OASM), adverse events (AIMS, BAS, SAS)	Lilly Research Laboratories
Ichikawa, 2017	Japan	Autism	DSM-IV-TR; PARS	CGI-S ≥ 4; ABC-I ≥ 18	Aripiprazole 1 to 15 mg/day, adaptive dose (47)	Placebo (45)	Parallel	8	Specialistic	10.1 (3.2); 6.0–17.0	18.5	IQ 20 to normal	Global changes, (CGI-S, CGAS), ABC, compulsion (CY-BOCS, compulsion scale only), adverse events (C-SSRS, AIMS, DIEPSS, BAS)	Otsuka Pharmaceutical Co., Ltd
Kent, 2013	USA	Autism	DSM-TR; ADI-R	CGI-S ≥ 4; ABC-I ≥ 18	Risperidone 0.125 to 0.175 mg/day, fixed dose (30); Risperidone 1.25 to 1.75 mg/day, fixed dose (31)	Placebo (35)	Parallel	32 (6 weeks DB treatment, 26 weeks open lable treatment)	Specialistic	9.0 (3.1); 5.0–17.0	13.0	MA ≥ 18 months	Global changes (CGI-S, CGI-I), ABC, compulsion (CY-BOCS), adverse events (EPS, SAS, BAS, AIMS)	Johnson & Johnson Pharmaceutical Research & Development, LLC
Loebel, 2016	USA	Autism	DSM-IV-TR; ADI-R	CGI-S ≥ 4; ABC-I ≥ 18	Lurasidone 20 mg/day, fixed dose (49); Lurasidone 60 mg/day, fixed dose (51)	Placebo (49)	Parallel	6	Specialistic	10.7 (3.0); 6.0–17.0	18.2	Not reported	Global changes (CGI-I, CGI-S), ABC, compulsion (CY-BOCS), caregiver strain (CGSQ), adverse events (AIMS, SAS, BAS)	Sunovion Pharmaceuticals, Inc
Luby, 2006	USA	ASD	DSM-IV	CARS ≥ 30	Risperidone 0.5 to 1.5 mg/day, adaptive dose (12)	Placebo (12)	Parallel	26	Specialistic	4.0 (1.9); 2.5–6.0	26.1	Not reported	CARS, GARS, adaptive behaviors (CBCL, VABS), communication (PLS-3)	Janssen Pharmaceutica
Marcus, 2009	USA	Autism	DSM-IV-TR; ADI-R	CGI-S ≥ 4; ABC-I ≥ 18	Aripiprazole 5 mg/day, fixed dose (53); Aripiprazole 10 mg/day, fixed dose (59); Aripiprazole 15 mg/day (54)	Placebo (52)	Parallel	8	Specialistic	9.7 (3.1), 6.0–17.0	10.6	MA ≥ 18 months	CGI-S, ABC, quality of life (PedsQL), compulsion (CY-BOCS), caregiver strain (CGSQ), adverse events (EPS, SAS, BAS, AIMS)	Bristol-Myers Squibb; Otsuka Pharmaceutical Co., Ltd
McCraken, 2002	USA	Autism	DSM-IV; ADI-R	CGI-S ≥ 4; ABC-I ≥ 18	Risperidone 0.25 to 2.5 (< 45 kg), 0.5 to 3.5 (≥ 45 kg) mg/day, adaptive dose (49)	Placebo (52)	Parallel	8	Specialistic	8.8 (2.7); 5.0–17.0	18.8	MA ≥ 18 months	Global changes (CGI-I), ABC, adverse events (SAS, AIMS)	NIMH; NIH; Korczak Foundation; Janssen Pharmaceutica
Nagaraj, 2006	India	Autism	DSM-IV	Not reported	Risperidone 1.0 mg/day, fixed dose (19)	Placebo (21)	Parallel	26	Specialistic	5.1 (1.7); 2.0–9.0	12.8	IQ ≥ 35	Global changes (CGAS), CARS, social quotient (VSMS), adverse events (AIMS)	Sun Pharmaceuticals
NCT00870727	USA	PDD-NOS	DSM-IV-TR	CGI-S ≥ 4; ABC-I ≥ 18	Aripiprazole 2.0 to 20.0 mg/ day, flexible dose (17)	Placebo (16)	Parallel	8	Specialistic	9.6 (2.7); 5.0–17.0	21.2	IQ ≥ 50	Global changes (CGI-I), ABC, adverse wevents	Indiana University; NIMH; Bristol-Myers Squibb
NCT01624675	Japan	Autism	DSM-IV-TR	CGI-S ≥ 4; ABC-I ≥ 18	Risperidone 1.0 (< 20 kg weight) or 2.5 (≥ 20 kg weight) mg/day (21)	Placebo (18)	Parallel	8	Specialistic	NR (NR); 5.0–17.0	NR	IQ ≥ 35; MA ≥ 18 months	Global changes (CGI-I, CGI-S, CGAS), ABC, parents satisfaction (PSQ), adverse events	Janssen Pharmaceutical K.K
Owen, 2009	USA	Autism	DSM-IV; ADI-R	CGI-S ≥ 4; ABC-I ≥ 18	Aripiprazole 2.0 to 15.0 mg/day, adaptive dose (47)	Placebo (51)	Parallel	8	Specialistic	9.3 (3.0); 6.0–17.0	12.2	MA ≥ 18 months	Global changes (CGI-I, CGI-S), ABC, compulsion (CY-BOCS), quality of life (PedsQL), caregiver strain (CGSQ), adverse events (AIMS, BAS, SAS)	Bristol-Myers Squibb; Otsuka Pharmaceutical Co, Ltd; Ogilvy Healthworld Medical Education
Remington, 2001	USA	Autism	DSM-IV	Not reported	Chlomipramine 100 to 150 mg/day (7); Haloperidol 1.0 to 1.5 mg/day (11)	Placebo (7)	Crossover	22 (1 weeks SB placebo, 21 weeks DB intervention, 3 weeks each one: clomipramine/placebo/haloperidol, placebo/haloperidol/clomipramine, and haloperidol/clomipramine/placebo)	Specialistic	12.8 (2.4); 10.0–17.0	12.0	Not reported	CARS, adverse events (DOTES, EPS)	Ontario Mental Health Foundation
RUPP, 2005	USA	Autism	DSM-IV; ADI-R	CGI-S ≥ 4; ABC-I ≥ 18	Risperidone 0.25 to 3.5 (< 45 kg), 0.5 to 4.5 (≥ 45 kg) mg/day, adaptive dose (16)	Placebo (16)	Withdrawal	8 (3 weeks DB taper, 5 weeks DB placebo)	Specialistic	9.0 (2.5); 5.0–17.0	13.2	Profound retardation to normal	Relapse (ABC, CGI-I)	NIMH; NIH; Korczak Foundation; Janssen Pharmaceutica
Shea, 2004	Canada	PDD	DSM-IV-TR; CARS	CARS ≥ 30	Risperidone 0.02 to 0.06 mg/kg/day, adaptive dose (41)	Placebo (39)	Parallel	8	Specialistic	7.5 (2.3); 5.0–12.0	22.8	IQ ≥ 35	Global change (CGI-C), ABC, VAS, behavior (N-CBRF), adverse events (ESRS)	Janssen-Ortho, Inc., Canada; Johnson & Johnson Pharmaceuticals
Troost, 2005	Netherlands	PDD (Autism, Asperger, PDD-NOS)	DSM-IV-TR; ADI-R	CGI-S ≥ 4; ABC-Irritability ≥ 18	Risperidone 0.5 to 6.0 mg/day, adaptive dose (12)	Placebo (12)	Withdrawal	8 (3 weeks DB taper, 5 weeks DB placebo)	Specialistic	9.1 (2.6); 5.0–17.0	8.3	MA ≥ 18 months	Relapse (ABC, CGI-S)	Not reported

Legend: ABC: Aberrant Behavior Checklist; ADI-R: Autism Diagnostic Interview—Revised; ADOS: Autism Diagnostic Observation Scale; BASC: Behavioral Assessment System for Children; CARS: Childhood Autism Rating Scale; CDI: Children's Depression Inventory; CGI-I: Clinical Global Impression-Improvement scale; CGI-S: Clinical Global Impression-Severity scale; DHA: docosahexaenoic acid; EPA: eicosapentaenoic acid; EVT: Expressive Vocabulary Test; NIH: National Institutes, of Health, U.S.; NIMH: National Institute of Mental Health, U.S.; PDDBI: Pervasive Developmental Disorder- Behavioral Inventory; PLS-4: Preschool Language Scale, Fourth Edition; PPVT: Peabody Picture Vocabulary Test; SCQ: Social Communication Questionnaire; SRS: Social Responsiveness Scale; VABS-II: Vineland Adaptive Behavior Scales, Second Edition

To see the results of risk of bias assessment of included studies, see Additional file 4. We assessed publication bias through funnel plots presented in the Additional file 7.

### Results and overall certainty of evidence

Forest plots for the main analyses are shown in Additional file 5, while the GRADE evidence profile is shown in Additional file 8.

Antipsychotics probably reduce “hyperactivity, attention deficit, opposition, and disruptive behaviors”, and probably slightly reduce both “restricted and repetitive interests and behaviors” and “obsessions, compulsions”.

Antipsychotics seem to slightly reduce “emotional dysregulation/irritability” and seem to positively influence “global functioning, global improvement”.

There are uncertainties about the effect of antipsychotics on “anxiety” and “self-harm”.

With regard to the safety profile, antipsychotics are likely to increase the risk of “adverse events”, and may induce a slight increase in the incidence of “serious adverse events”.

We found no extractable data regarding “insomnia” and “quality of life” outcomes.

Details about effect estimates and certainty of evidence are reported in Tables 2 and 3: Summary of Findings tables.

**Table 2:** Summary of Findings (SoF) for the comparison antipsychotics versus placebo – continuous outcomes

Antipsychotics versus no antipsychotics – continuous outcomes						
Population: children and adolescents with ASD Setting: outpatients and inpatients Intervention: antipsychotics Comparator: no antipsychotics						
Outcomes	Anticipated absolute effects^*^ (95% CI)		Relative effect (95% CI)	№ of participants (studies)	Certainty of the evidence (GRADE)	Comments
	Risk with no Antipsychotics	Risk with Antipsychotics				
Restricted and repetitive interests and behaviors	–	SMD 0.21 SD lower (0.35 lower to 0.07 lower)	–	823 (9 RCTs)	⨁⨁⨁◯ MODERATE ^a^	Antipsychotics probably slightly reduce restricted and repetitive interests and behaviors
Hyperactivity, inattention, oppositional, disruptive behavior	–	SMD 0.67 SD lower (0.92 lower to 0.42 lower)	–	783 (8 RCTs)	⨁⨁⨁◯ MODERATE ^a^	Antipsychotics probably reduce Hyperactivity, inattention, oppositional, disruptive behavior disorders
Self-harm	–	SMD 0.14 SD lower (0.58 lower to 0.30 higher)	–	77 (1 RCT)	⨁◯◯◯ VERY LOW ^b,c^	There are some uncertainties about the effect of Antipsychotics on self-harm
Social communication, social interaction	–	SMD 0.38 SD lower (0.59 lower to 0.16 lower)	–	854 (10 RCTs)	⨁⨁⨁◯ MODERATE ^a^	Antipsychotics probably slightly improve social communication, social interaction
Emotional dysregulation/irritability	–	SMD 0.71 SD lower (0.98 lower to 0.43 lower)	–	879 (9 RCTs)	⨁⨁◯◯ LOW ^a,d^	Antipsychotics administration may result in a large reduction of emotional dysregulation/irritability
Anxiety	–	SMD 0.38 SD lower (0.82 lower to 0.07 higher)	–	77 (1 RCT)	⨁◯◯◯ VERY LOW ^b,c^	There are some uncertainties about the effect of Antipsychotics on anxiety
Global functioning, global improvement	–	SMD 0.64 SD lower (0.96 lower to 0.33 lower)	–	839 (10 RCTs)	⨁⨁◯◯ LOW ^e,f^	Antipsychotics may influence positively Global functioning, global improvement
Obsessions, compulsions	–	SMD 0.30 SD lower (0.55 lower to 0.06 lower)	–	548 (4 RCTs)	⨁⨁⨁◯ MODERATE ^g^	Antipsychotics probably slightly reduce obsessions, compulsions

*The risk in the intervention group (and its 95% confidence interval) is based on the assumed risk in the comparison group and the relative effect of the intervention (and its 95% CI). CI: Confidence interval; SMD: Standardised mean difference; RR: Risk ratio

GRADE Working Group grades of evidence High certainty: We are very confident that the true effect lies close to that of the estimate of the effect Moderate certainty: We are moderately confident in the effect estimate: The true effect is likely to be close to the estimate of the effect, but there is a possibility that it is substantially different Low certainty: Our confidence in the effect estimate is limited: The true effect may be substantially different from the estimate of the effect Very low certainty: We have very little confidence in the effect estimate: The true effect is likely to be substantially different from the estimate of effect

Explanations

a. Downgraded by one level because most studies showed an unclear risk of bias for selection bias and one study was at high risk for attrition bias

b. Downgraded by one level because the included study was at high risk for selection bias

c. Downgraded by two levels because the sample size is very small (< 400) and the 95%CI for SMD goes from considerable beneficial effects to considerable undesirable effects

d. Downgraded by one level for heterogeneity (I^2^ = 71.0%), several confidence intervals of the included trial not overlapping and important differences in the point estimates

e. Downgraded by one level because most studies showed an unclear risk for selection bias, two studies were at high risk for reporting bias and one study was at high risk for selection bias

f. Downgraded by one level for heterogeneity (I^2^ = 75.6%), several confidence intervals of the included trial not overlapping and important differences in the point estimates

g. Downgraded by one level because the 95%CI for SMD goes from considerable beneficial effects to not clinically relevant effects

**Table 3:** Summary of Findings (SoF) for the comparison antipsychotics versus placebo –dichotomous outcomes

Antipsychotics versus no antipsychotics – dichotomous outcomes						
Population: children and adolescents with ASD Setting: outpatients and inpatients Intervention: Antipsychotics Comparator: no Antipsychotics						
Outcomes	Anticipated absolute effects^*^ (95% CI)		Relative effect (95% CI)	№ of participants (studies)	Certainty of the evidence (GRADE)	Comments
	Risk with no Antipsychotics	Risk with Antipsychotics				
Serious adverse events	16 per 1.000	17 per 1.000 (8 to 39)	RR 1.07 (0.48 a 2.43)	1057 (13 RCTs)	⨁⨁◯◯ LOW ^a,b^	Antipsychotics may increase the risk of severe adverse events slightly
Adverse events	657 per 1.000	781 per 1.000 (703 to 867)	RR 1.19 (1.07 a 1.32)	924 (10 RCTs)	⨁⨁⨁◯ MODERATE ^c^	Antipsychotics probably increase the risk of adverse events
Dropout due to any cause	244 per 1.000	149 per 1.000 (117 to 190)	RR 0.61 (0.48 a 0.78)	1124 (15 RCTs)	⨁⨁⨁◯ MODERATE ^d^	Antipsychotics probably reduce the risk of dropout due to any cause
Drop-out due to adverse events	39 per 1.000	39 per 1.000 (22 to 70)	RR 0.99 (0.55 to 1.79)	1010 (12 RCTs)	⨁⨁◯◯ LOW ^b,e^	Antipsychotics may have little or no effect on the risk of dropouts due to adverse events

*The risk in the intervention group (and its 95% confidence interval) is based on the assumed risk in the comparison group and the relative effect of the intervention (and its 95% CI). **CI:** Confidence interval; SMD: Standardised mean difference; RR: Risk ratio

GRADE Working Group grades of evidence High certainty: We are very confident that the true effect lies close to that of the estimate of the effect

Moderate certainty: We are moderately confident in the effect estimate: The true effect is likely to be close to the estimate of the effect, but there is a possibility that it is substantially different

Low certainty: Our confidence in the effect estimate is limited: The true effect may be substantially different from the estimate of the effect

Very low certainty: We have very little confidence in the effect estimate: The true effect is likely to be substantially different from the estimate of effect

Explanations

a. Downgraded by one level because most studies showed an unclear risk for selection bias, three studies were at high risk for attrition bias, one study was at high risk for selection bias and one study was at high risk for reporting bias

b. Downgraded by one level because the 95%CI for SMD goes from considerable beneficial effects to considerable undesirable effects

c. Downgraded by one level because most studies showed an unclear risk of bias for selection bias and two studies were at high risk for attrition bias

d. Downgraded by one level because most studies showed an unclear risk for selection bias, four studies were at high risk for attrition bias, one study was at high risk for selection bias and one study was at high risk for reporting bias

e. Downgraded by one level because most studies showed an unclear risk for selection bias, four studies were at high risk for attrition bias, one was at high risk for selection bias and one was at high risk for reporting bias

### Subgroup effect considerations

Antipsychotics in the subgroup with ABC-Irritability ≥ 18 showed a trend towards a higher efficacy for emotional dysregulation and global functioning, and a trend towards lower efficacy for social communication, social interaction and hyperactivity outcomes. In the same subgroup antipsychotics apparently showed a better safety and tolerability profile (see Additional File 6). However, when considering whether a clear subgroup effect was present or not, we noted that: summary effects for the subgroup versus all were overlapping; nearly all 95% CIs of individual studies were overlapping for all outcomes; there was not a clear suggestion of consistently greater benefits or harms in the subgroup; the p-values for subgroup effects indicated that differences were likely due to chance.

We considered that there were no subgroup effects that are substantially concerning or credible, and certainty of evidence was not downgraded for subgroup effects.

## Discussion

We found antipsychotics for children and adolescents with ASD more efficacious, more acceptable, but less safe than placebo.

Antipsychotics are often used as off-label pharmacological treatments for children and adolescents, even though their use in this population is an evidence-based choice for certain conditions [111]. According to a meta-analysis conducted in 2017 by Pillay et al. [13], and similarly to what we found in our review, SGAs were probably effective in reducing irritability, and in producing a slight decrease in social withdrawal, stereotypy and inappropriate speech, as measured by ABC. SGAs also probably increased response rate and decreased global impression of severity. Recent meta-analyses comparing only one drug (i.e. risperidone, aripiprazole, and lurasidone) versus placebo showed all similar results, even if more often did not report significant estimates, probably due to wider confidence intervals, particularly for lurasidone [112–114]. Pillay et al. [13] described narratively head-to-head trials, reporting (1) no differences between aripiprazole and risperidone in main outcomes, (2) mixed results when comparing haloperidol to risperidone or olanzapine. Since the publication of Pillay et al. (2017) systematic review, results from 4 placebo controlled [77, 95, 100, 102] and 2 head-to-head [53, 83] RCTs have been published.

In our study, antipsychotics in short- and medium-term showed good tolerability, as they reduced the risk of dropouts by 39% (moderate certainty), and demonstrated to be relatively safe options, with an increase of 19% in adverse events (moderate certainty) and 9% in serious/severe adverse events (low certainty). Evaluating the long-term safety profile of the antipsychotics was not among the objectives of this systematic review, as we did not perform a systematic review of not-randomized studies. However, it can be stated that, in children and adolescents with ASD: long-term use of risperidone, although generally well-tolerated, has been associated with an increase in plasma glucose, insulin, prolactin and leptin proportional to the dosage; the major problems often resulted from continuous weight gain over time and judged excessive [115–117]; the continued use of aripiprazole has been associated with a decrease in prolactinemia, with a similar risk profile for weight gain [118, 119]. The transition of treatment from risperidone to aripiprazole seems to reduce adverse events such as drowsiness, hyper-prolactinemia and amenorrhoea [120]. The number needed to treat (NNT) evaluated for effectiveness on irritability was lower using risperidone than aripiprazole, but on the other hand the number needed to harm (NNH) for the onset of extrapyramidal symptoms was also lower with risperidone [8]. The use of antipsychotics in general has also been related to hyperuricemia and hyperprolactinemia [121–123].

### Study limitations

We have not prospectively registered the protocol for our systematic review; however, the clinical question was formulated by a multidisciplinary panel of experts, and we followed the methodology reported in the manual developed and published by the ISS [14].

After the systematic review was performed and the recommendation formulated by the panel was submitted to public consultation (https://www.osservatorionazionaleautismo.it/attivita-istituzionali/linee-guida/consultazioni-pubbliche), the results of a previously ongoing trial (NCT00198107) [77] were published on clinicaltrial.gov: 40 individuals were randomized to aripiprazole and 41 to placebo for 8 weeks. Data were available for “restricted and repetitive interests and behavior”, “Hyperactivity, inattention, oppositional, disruptive behavior disorders”, “Social communication, social interaction”, “Emotional dysregulation/irritability” (ABC subscales), for “obsessions, compulsions” (CY-BOCS), for “global functioning, global improvement” (ADOS), and for safety and tolerability outcomes (adverse events, dropouts and serious adverse events). Outcome data were consistent with what we found in our meta-analyses; for all these outcomes, except for emotional dysregulation/irritability and global functioning, the certainty of evidence was already rated as moderate. The Evidence Review Team, together with the content expert and the panel chair, considered that the new findings did not change the body of evidence and that there was no need to carry out new statistical analyses nor to reformulate the recommendation for this intervention.

The main limitation in the subgroup analysis was that the “all ASDs” studies included a mixed population, while ideally, we would have had groups with and without problem behaviors.

Finally, the use of the EtD framework requires the panel to be familiar with the tool [124]. To overcome this potential limitation, about 2 months before the presentation of the body of evidence on antipsychotics, an EtD framework on a pilot question was presented to the panel [125, 126] for the formulation of a recommendation on the use of polyunsaturated fatty acids. This recommendation will not take part to the Italian guidelines on the diagnosis and treatment of children and adolescents with ASD. In other experiences, panel members have reported that, when familiarity with the EtD framework is achieved, the tool helped them in structuring discussion, saving time, ensuring systematicity in the process of recommendation formulation [127].

## Conclusions

We found antipsychotics in children and adolescents with ASD to be significantly more efficacious than placebo in reducing stereotypies, hyperactivity, irritability and obsessions, compulsions, and in increasing social communication and global functioning. Antipsychotics were also found to be significantly more acceptable in terms of dropouts due to any cause, but significantly less safe in terms of patients experiencing adverse events. We found no evidence regarding the impact of antipsychotics on “insomnia” and “quality of life” outcomes in this population. Available evidence on efficacy and safety of antipsychotics in children and adolescents with ASD needs to be evaluated together with evidence on equity, acceptability, feasibility [28], resources required and cost-effectiveness [29] in formulating a recommendation.

Preferred reporting items for systematic reviews and meta-analyses (PRISMA) checklist is reported on Additional file 9.

## Supplementary information

**Additional file 1:** Search strategy and results for Systematic Reviews.

**Additional file 2:** Search strategy and results for Randomized Controlled Trials.

**Additional file 3:** References for included and excluded trials, with reasons.

**Additional file 4:** Risk of Bias Summary..

**Additional file 5:** Forest plots for comparisons between antipsychotics (D2 blockers) and Placebo.

**Additional file 6:** Forest plots for comparisons between antipsychotics (D2 blockers) and Placebo – subgroup analyses (ABC-Irritability ≥18 vs All ASDs).

**Additional file 7:** Funnel Plots for outcome with estimates for at least 8 studies.

**Additional file 8:** GRADE Evidence profile.

**Additional file 9:** PRISMA checklist.

## Abbreviations

ABC: Aberrant Behavior Checklist
ADHD: attention deficit hyperactivity disorder
ASD: autism spectrum disorder
BASC: behavior assessment system for children
CGI-I: Clinical Global Impression-Improvement scale
CGI-S: Clinical Global Impression-Severity Scale
CI: confidence interval
EtD: evidence to decision
EVT: expressive vocabulary test
FGA: first generation antipsychotics
GRADE: Grading of Recommendations Assessment, Development and Evaluation
ISS: Istituto Superiore di Sanità (Italian National Institute of Health)
MSEL: Mullen Scales of Early Learning
PLS: The Preschool Language Scale
PPVT: Peabody Picture Vocabulary Test
RCT: randomized controlled trial
RR: risk ratio
SGA: Second Generation Antipsychotics
SMD: Standardized mean difference
SRS: Social Responsiveness Scale
VABS: Vineland Adaptive Behavior Scale

## Availability of data and materials

All data supporting our findings is contained within the manuscript and the additional files.
