# Supplementary material for: Impact of antipsychotics in children and adolescents with autism spectrum disorder: a systematic review and meta-analysis
Source: Health Qual Life Outcomes. 2021 Jan 25;19:33. doi: 10.1186/s12955-021-01669-0 (PMC7831175; doi:10.1186/s12955-021-01669-0)
Supplement: Supplementary file 1 — Additional file 1: Search strategy and results for Systematic Reviews. [file 12955_2021_1669_MOESM1_ESM.docx]

Additional file 1.

# Search strategy and results for Systematic Reviews

## Cochrane Database of Systematic Reviews (CDSR) search strategy

**Cochrane Database of Systematic Reviews (Issue 12, 2018)**

#1 MeSH descriptor: [Child Development Disorders, Pervasive] explode all trees

#2 asperger*

#3 (autis* or ASD or ASDs):ti,ab,kw

#4 kanner*

#5 ((pervasiv* NEXT development* NEXT disorder*) OR PDD or PDDs):ti,ab,kw

#6 #1 or #2 or #3 or #4 # or #5 4296

#7 MeSH descriptor: [Antipsychotic Agents] explode all trees

#8 MeSH descriptor: [Butyrophenones] explode all trees

#9 MeSH descriptor: [Phenothiazines] 2 tree(s) exploded

#10 MeSH descriptor: [Thioxanthenes] explode all trees

#11 (anti next psychotic*) or antipsychotic*:ti,ab,kw

#12 Amisulprid*

#13 Aripiprazol*

#14 Asenapin*

#15 Blonanserin

#16 Chlorpromazin*

#17 Chlorprothixen*

#18 Clotiapin*

#19 Clozapin*

#20 Droperidol

#21 Flupentixol

#22 Fluphenazin*

#23 Haloperidol

#24 Iloperidon*

#25 Levomepromazin*

#26 Loxapin*

#27 Lurasidon*

#28 Melperon*

#29 Mesoridazin*

#30 Molindon*

#31 Mosapramin*

#32 Olanzapin*

#33 Paliperidon*

#34 Periciazin*

#35 Perospiron*

#36 Pimozid*

#37 Prochlorperazin*

#38 Promazin*

#39 Quetiapin*

#40 Remoxiprid*

#41 Risperidon*

#42 Sertindol*

#43 Sulpirid*

#44 Thioproperazin*

#45 Thioridazin*

#46 Thiothixen*

#47 Tiaprid*

#48 Trifluoperazin*

#49 Ziprasidon*

#50 Zotepin*

#51 Zuclopenthixol

#52 {or #7-#51}

#53 #6 and #52

#54 MeSH descriptor: [Adolescent] explode all trees

#55 MeSH descriptor: [Adolescent Medicine] explode all trees

#56 MeSH descriptor: [Child] explode all trees

#57 MeSH descriptor: [Minors] explode all trees

#58 MeSH descriptor: [Pediatrics] explode all trees

#59 MeSH descriptor: [Young Adult] explode all trees

#60 (child* or schoolchild* or kid or kids or toddler* or adoles* or teen*or boy* or girl* or minors* or underag* or under age or juvenil* or youth* or kindergar* or puberty or pubescen* or prepubescen* or prepuberty* or pediatric* or paediatric* or peadiatric* or preschool* or schoolage):ti,ab,kw

#61 ((grade next school*) or (pre next school*) or (school next age*) or schoolchild*):ti,ab,kw

#62 ((colleg* or highschool* or school* or universit*) near/2 (age* or student*)):ti,ab,kw

#63 (young* next (adult* or men or mens or people* or person* or women*))

#64 #54 or #55 or #56 or #57 or #58 or #59 or #60 or #61 or #62 or #63

#65 #53 AND #64

## MEDLINE search strategy

**Ovid MEDLINE (Ovid MEDLINE® Epub Ahead of Print, In-Process & Other Non-Indexed Citations, Ovid MEDLINE® Daily and Ovid MEDLINE®), 1946 to December 16, 2018**

1 exp Child Development Disorders, Pervasive/

2 (autis* or ASD or ASDs or PDD or PDDs).tw.

3 pervasive developmental disorder$.tw.

4 asperg$.tw.

5 kanner$.tw.

6 1 or 2 or 3 or 4 or 5

7 exp child/ or adolescent/ or pediatrics/

8 (child* or schoolchild* or kid or kids or toddler* or adoles* or teen*or boy* or girl* or minors* or underag* or under age or juvenil* or youth* or kindergar* or puberty or pubescen* or prepubescen* or prepuberty* or pediatric* or paediatric* or peadiatric* or preschool* or schoolage).tw.

9 (school adj2 age*).ti,ab.

10 7 or 8 or 9

11 6 and 10

12 exp Antipsychotic Agents/

13 exp BUTYROPHENONES/

14 exp PHENOTHIAZINES/

15 exp THIOXANTHENES/

16 (anti psychotic* or antipsychotic*).mp.

17 amisulprid$.mp.

18 aripiprazol*.mp.

19 asenapin*.mp.

20 Blonanserin.mp.

21 Chlorpromazin*.mp.

22 Chlorprothixen*.mp.

23 Clotiapin*.mp.

24 Clozapin*.mp.

25 Droperidol.mp.

26 Flupentixol.mp.

27 Fluphenazin*.mp.

28 Haloperidol.mp.

29 Iloperidon*.mp.

30 Levomepromazin*.mp.

31 Loxapin*.mp.

32 Lurasidon*.mp.

33 Melperon*.mp.

34 Mesoridazin*.mp.

35 Molindon*.mp.

36 Mosapramin*.mp.

37 Olanzapin*.mp.

38 Paliperidon*.mp.

39 Periciazin*.mp.

40 Perospiron*.mp.

41 Pimozid*.mp.

42 Prochlorperazin*.mp.

43 Promazin*.mp.

44 Quetiapin*.mp.

45 Remoxiprid*.mp.

46 Risperidon*.mp.

47 Sertindol*.mp.

48 Sulpirid*.mp.

49 Thioproperazin*.mp.

50 Thioridazin*.mp.

51 Thiothixen*.mp.

52 Tiaprid*.mp.

53 Trifluoperazin*.mp.

54 Ziprasidon*.mp.

55 Zotepin*.mp.

56 Zuclopenthixol.mp.

57 Mesoridazin*.mp.

58 12 or 13 or 14 or 15 or 16 or 17 or 18 or 19 or 20 or 21 or 22 or 23 or 24 or 25 or 26 or 27 or 28 or 29 or 30 or 31 or 32 or 33 or 34 or 35 or 36 or 37 or 38 or 39 or 40 or 41 or 42 or 43 or 44 or 45 or 46 or 47 or 48 or 49 or 50 or 51 or 52 or 53 or 54 or 55 or 56 or 57

59 11 and 58

60 meta-analysis/ or systematic review/ or meta-analysis as topic/ or "meta analysis (topic)"/ or "systematic review (topic)"/ or exp technology assessment, biomedical/

61 ((systematic* adj3 (review* or overview*)) or (methodologic* adj3 (review* or overview*))).ti,ab,kf,kw.

62 ((quantitative adj3 (review* or overview* or synthes*)) or (research adj3 (integrati* or overview*))).ti,ab,kf,kw.

63 ((integrative adj3 (review* or overview*)) or (collaborative adj3 (review* or overview*)) or (pool* adj3 analy*)).ti,ab,kf,kw.

64 (data synthes* or data extraction* or data abstraction*).ti,ab,kf,kw.

65 (handsearch* or hand search*).ti,ab,kf,kw.

66 (handsearch* or hand search*).ti,ab,kf,kw.

67 (meta regression* or metaregression*).ti,ab,kf,kw.

68 (meta-analy* or metaanaly* or systematic review* or biomedical technology assessment* or bio-medical technology assessment*).mp,hw.

69 (medline or cochrane or pubmed or medlars or embase or cinahl).ti,ab,hw.

70 (cochrane or (health adj2 technology assessment) or evidence report).jw.

71 (comparative adj3 (efficacy or effectiveness)).ti,ab,kf,kw.

72 (outcomes research or relative effectiveness).ti,ab,kf,kw.

73 ((indirect or indirect treatment or mixed-treatment) adj comparison*).ti,ab,kf,kw.

74 60 or 61 or 62 or 63 or 64 or 65 or 66 or 67 or 68 or 69 or 70 or 71 or 72 or 73

75 59 and 74

## EMBASE search strategy

**EMBASE (via embase.com)**

**Database: Embase Classic + Embase, 1947 to 2018 December 16**

1 exp autism/

2 (autis* or ASD or ASDs or PDD or PDDs).tw.

3 pervasive developmental disorder$.tw.

4 asperg$.tw.

5 kanner$.tw.

6 1 or 2 or 3 or 4 or 5

7 exp child/ or adolescent/ or pediatrics/

8 (child* or schoolchild* or kid or kids or toddler* or adoles* or teen*or boy* or girl* or minors* or underag* or under age or juvenil* or youth* or kindergar* or puberty or pubescen* or prepubescen* or prepuberty* or pediatric* or paediatric* or peadiatric* or preschool* or schoolage).tw.

9 (school adj2 age*).ti,ab.

10 7 or 8 or 9

11 6 and 10

12 exp neuroleptic agent/

13 (anti psychotic* or antipsychotic*).mp.

14 amisulprid$.mp.

15 aripiprazol*.mp.

16 asenapin*.mp.

17 Blonanserin.mp.

18 Chlorpromazin*.mp.

19 Chlorprothixen*.mp.

20 Clotiapin*.mp.

21 Clozapin*.mp.

22 Droperidol.mp.

23 Flupentixol.mp.

24 Fluphenazin*.mp.

25 Haloperidol.mp.

26 Iloperidon*.mp.

27 Levomepromazin*.mp.

28 Loxapin*.mp.

29 Lurasidon*.mp.

30 Melperon*.mp.

31 Mesoridazin*.mp.

32 Molindon*.mp.

33 Mosapramin*.mp.

34 Olanzapin*.mp.

35 Paliperidon*.mp.

36 Periciazin*.mp.

37 Perospiron*.mp.

38 Pimozid*.mp.

39 Prochlorperazin*.mp.

40 Promazin*.mp.

41 Quetiapin*.mp.

42 Remoxiprid*.mp.

43 Risperidon*.mp.

44 Sertindol*.mp.

45 Sulpirid*.mp.

46 Thioproperazin*.mp.

47 Thioridazin*.mp.

48 Thiothixen*.mp.

49 Tiaprid*.mp.

50 Trifluoperazin*.mp.

51 Ziprasidon*.mp.

52 Zotepin*.mp.

53 Zuclopenthixol.mp.

54 Mesoridazin*.mp.

55 12 or 13 or 14 or 15 or 16 or 17 or 18 or 19 or 20 or 21 or 22 or 23 or 24 or 25 or 26 or 27 or 28 or 29 or 30 or 31 or 32 or 33 or 34 or 35 or 36 or 37 or 38 or 39 or 40 or 41 or 42 or 43 or 44 or 45 or 46 or 47 or 48 or 49 or 50 or 51 or 52 or 53 or 54

56 11 and 55

57 "systematic review"/ or meta analysis/

58 "meta analysis (topic)"/

59 "systematic review (topic)"/

60 biomedical technology assessment/

61 ((systematic* adj3 (review* or overview*)) or (methodologic* adj3 (review* or overview*))).ti,ab.

62 ((quantitative adj3 (review* or overview* or synthes*)) or (research adj3 (integrati* or overview*))).ti,ab.

63 ((integrative adj3 (review* or overview*)) or (collaborative adj3 (review* or overview*)) or (pool* adj3 analy*)).ti,ab.

64 (data synthes* or data extraction* or data abstraction*).ti,ab.

65 (handsearch* or hand search*).ti,ab.

66 (mantel haenszel or peto or der simonian or dersimonian or fixed effect* or latin square*).ti,ab.

67 (met analy* or metanaly* or technology assessment* or HTA or HTAs or technology overview* or technology appraisal*).ti,ab.

68 (meta regression* or metaregression*).ti,ab.

69 (meta-analy* or metaanaly* or systematic review* or biomedical technology assessment* or bio-medical technology assessment*).mp,hw.

70 (medline or cochrane or pubmed or medlars or embase or cinahl).ti,ab.

71 (cochrane or (health adj2 technology assessment) or evidence report).jw.

72 (comparative adj3 (efficacy or effectiveness)).ti,ab.

73 (outcomes research or relative effectiveness).ti,ab.

74 ((indirect or indirect treatment or mixed-treatment) adj comparison*).ti,ab.

75 57 or 58 or 59 or 60 or 61 or 62 or 63 or 64 or 65 or 66 or 67 or 68 or 69 or 70 or 71 or 72 or 73 or 74

76 56 and 75

## Numbers of citations by each database

| **Databases, trial registers and other sources** | **Citations** |
| --- | --- |
| **Databases:** |  |
| MEDLINE | 78 |
| Cochrane Database of Systematic Reviews (CDSR) | 13 |
| Embase | 192 |
| **Total (databases)** | **283** |
| Duplicate records removed | 63 |
| **Total (databases) after duplicates removed** | **220** |

# Systematic Reviews evaluated in full-text, with reasons for exclusion

## Excluded Systematic Reviews, with reasons

We retrieved from database searching 220 citations, of which 7 were considered potentially eligible to provide an answer to our question. However, we excluded all of these systematic reviews for the following reasons: only one drug considered (Channing 2018, Maneeton 2018a, Maneeton 2018b, Hirsch 2016), meta-analyses not performed (Channng 2018, Lee 2018), wrong study population (Channing 2018, Lee 2018); too old update of the search strategy (older than 18 months) (Hirsch 2016, Pillay 2017), several outcomes of interest not included, some interventions not included in the analyses (Fallah 2019).

## References for excluded Systematic Reviews

• Channing J, Mitchell M, Cortese S. Lurasidone in Children and Adolescents: Systematic Review and Case Report. J Child Adolesc Psychopharmacol. 2018 Sep;28(7):428-436. doi: 10.1089/cap.2018.0046. Epub 2018 Jul 13. PubMed PMID: 30004236.

• Hirsch LE, Pringsheim T. Aripiprazole for autism spectrum disorders (ASD). Cochrane Database Syst Rev. 2016 Jun 26;(6):CD009043. doi:10.1002/14651858.CD009043.pub3. Review. PubMed PMID: 27344135.

• Fallah MS, Shaikh MR, Neupane B, Rusiecki D, Bennett TA, Beyene J. Atypical

Antipsychotics for Irritability in Pediatric Autism: A Systematic Review and

Network Meta-Analysis. J Child Adolesc Psychopharmacol. 2019 Apr;29(3):168-180.

doi: 10.1089/cap.2018.0115. Epub 2019 Feb 1. PubMed PMID: 30707602.

• Lee ES, Vidal C, Findling RL. A Focused Review on the Treatment of Pediatric Patients with Atypical Antipsychotics. J Child Adolesc Psychopharmacol. 2018 Nov;28(9):582-605. doi: 10.1089/cap.2018.0037. Epub 2018 Oct 12. Review. PubMed PMID: 30312108.

• Maneeton N, Maneeton B, Putthisri S, Suttajit S, Likhitsathian S, Srisurapanont M. Aripiprazole in acute treatment of children and adolescents with autism spectrum disorder: a systematic review and meta-analysis. Neuropsychiatr Dis Treat. 2018 Nov 12;14:3063-3072. doi: 10.2147/NDT.S174622. eCollection 2018. PubMed PMID: 30519027; PubMed Central PMCID: PMC6237245.

• Maneeton N, Maneeton B, Putthisri S, Woottiluk P, Narkpongphun A, Srisurapanont M. Risperidone for children and adolescents with autism spectrum disorder: a systematic review. Neuropsychiatr Dis Treat. 2018 Jul 11;14:1811-1820. doi: 10.2147/NDT.S151802. eCollection 2018. PubMed PMID:30022830; PubMed Central PMCID: PMC6045903.

• Pillay J, Boylan K, Carrey N, Newton A, Vandermeer B, Nuspl M, MacGregor T, Jafri SHA, Featherstone R, Hartling L. First- and Second-Generation Antipsychotics in Children and Young Adults: Systematic Review Update [Internet]. Rockville (MD): Agency for Healthcare Research and Quality (US); 2017 Mar. Available from http://www.ncbi.nlm.nih.gov/books/NBK442352/PubMed PMID: 28749632.
