# Supplementary material for: Impact of antipsychotics in children and adolescents with autism spectrum disorder: a systematic review and meta-analysis
Source: Health Qual Life Outcomes. 2021 Jan 25;19:33. doi: 10.1186/s12955-021-01669-0 (PMC7831175; doi:10.1186/s12955-021-01669-0)
Supplement: Supplementary file 2 — Additional file 2: Search strategy and results for Randomized Controlled Trials. [file 12955_2021_1669_MOESM2_ESM.docx]

Additional file 2.

**Search Strategy and Results for Randomized Controlled Trials.**

# 1. CENTRAL search strategy

**CENTRAL (via onlinelibrary.wiley.com)**

1. MeSH descriptor: [Child Development Disorders, Pervasive] explode all trees
2. asperger*
3. (autis* or ASD or ASDs):ti,ab,kw
4. kanner*
5. ((pervasiv* NEXT development* NEXT disorder*) OR PDD or PDDs):ti,ab,kw
6. #1 or #2 or #3 or #4 # or #5
7. MeSH descriptor: [Antipsychotic Agents] explode all trees
8. MeSH descriptor: [Butyrophenones] explode all trees
9. MeSH descriptor: [Phenothiazines] explode all trees
10. MeSH descriptor: [Thioxanthenes] explode all trees
11. (anti next psychotic*) or antipsychotic*:ti,ab,kw
12. Amisulprid*
13. Aripiprazol*
14. Asenapin*
15. Blonanserin
16. Chlorpromazin*
17. Chlorprothixen*
18. Clotiapin*
19. Clozapin*
20. Droperidol
21. Flupentixol
22. Fluphenazin*
23. Haloperidol
24. Iloperidon*
25. Levomepromazin*
26. Loxapin*
27. Lurasidon*
28. Melperon*
29. Mesoridazin*
30. Molindon*
31. Mosapramin*
32. Olanzapin*
33. Paliperidon*
34. Periciazin*
35. Perospiron*
36. Pimozid*
37. Prochlorperazin*
38. Promazin*
39. Quetiapin*
40. Remoxiprid*
41. Risperidon*
42. Sertindol*
43. Sulpirid*
44. Thioproperazin*
45. Thioridazin*
46. Thiothixen*
47. Tiaprid*
48. Trifluoperazin*
49. Ziprasidon*
50. Zotepin*
51. Zuclopenthixol
52. {or #7-#51}
53. #6 and #52
54. MeSH descriptor: [Adolescent] explode all trees
55. MeSH descriptor: [Adolescent Medicine] explode all trees
56. MeSH descriptor: [Child] explode all trees
57. MeSH descriptor: [Minors] explode all trees
58. MeSH descriptor: [Pediatrics] explode all trees
59. MeSH descriptor: [Young Adult] explode all trees
60. (child* or schoolchild* or kid or kids or toddler* or adoles* or teen*or boy* or girl* or minors* or underag* or under age or juvenil* or youth* or kindergar* or puberty or pubescen* or prepubescen* or prepuberty* or pediatric* or paediatric* or peadiatric* or preschool* or schoolage):ti,ab,kw
61. ((grade next school*) or (pre next school*) or (school next age*) or schoolchild*):ti,ab,kw
62. ((colleg* or highschool* or school* or universit*) near/2 (age* or student*)):ti,ab,kw
63. (young* next (adult* or men or mens or people* or person* or women*))
64. #54 or #55 or #56 or #57 or #58 or #59 or #60 or #61 or #62 or #63
65. #53 AND #64

# 2. MEDLINE search strategy

**MEDLINE (Ovid MEDLINE® Epub Ahead of Print, In-Process & Other Non-Indexed Citations, Ovid MEDLINE® Daily and Ovid MEDLINE®) 1946 to January 22, 2019**

1. exp Child Development Disorders, Pervasive/
2. (autis* or ASD or ASDs or PDD or PDDs).tw.
3. pervasive developmental disorder$.tw.
4. asperg$.tw.
5. kanner$.tw.
6. 1 or 2 or 3 or 4 or 5
7. exp child/ or adolescent/ or pediatrics/
8. (child* or schoolchild* or kid or kids or toddler* or adoles* or teen*or boy* or girl* or minors* or underag* or under age or juvenil* or youth* or kindergar* or puberty or pubescen* or prepubescen* or prepuberty* or pediatric* or paediatric* or peadiatric* or preschool* or schoolage).tw.
9. (school adj2 age*).ti,ab.
10. 7 or 8 or 9
11. 6 and 10
12. exp Antipsychotic Agents/
13. exp BUTYROPHENONES/
14. exp PHENOTHIAZINES/
15. exp THIOXANTHENES/
16. (anti psychotic* or antipsychotic*).mp.
17. amisulprid$.mp.
18. aripiprazol*.mp.
19. asenapin*.mp.
20. Blonanserin.mp.
21. Chlorpromazin*.mp.
22. Chlorprothixen*.mp.
23. Clotiapin*.mp.
24. Clozapin*.mp.
25. Droperidol.mp.
26. Flupentixol.mp.
27. Fluphenazin*.mp.
28. Haloperidol.mp.
29. Iloperidon*.mp.
30. Levomepromazin*.mp.
31. Loxapin*.mp.
32. Lurasidon*.mp.
33. Melperon*.mp.
34. Mesoridazin*.mp.
35. Molindon*.mp.
36. Mosapramin*.mp.
37. Olanzapin*.mp.
38. Paliperidon*.mp.
39. Periciazin*.mp.
40. Perospiron*.mp.
41. Pimozid*.mp.
42. Prochlorperazin*.mp.
43. Promazin*.mp.
44. Quetiapin*.mp.
45. Remoxiprid*.mp.
46. Risperidon*.mp.
47. Sertindol*.mp.
48. Sulpirid*.mp.
49. Thioproperazin*.mp.
50. Thioridazin*.mp.
51. Thiothixen*.mp.
52. Tiaprid*.mp.
53. Trifluoperazin*.mp.
54. Ziprasidon*.mp.
55. Zotepin*.mp.
56. Zuclopenthixol.mp.
57. Mesoridazin*.mp.
58. 12 or 13 or 14 or 15 or 16 or 17 or 18 or 19 or 20 or 21 or 22 or 23 or 24 or 25 or 26 or 27 or 28 or 29 or 30 or 31 or 32 or 33 or 34 or 35 or 36 or 37 or 38 or 39 or 40 or 41 or 42 or 43 or 44 or 45 or 46 or 47 or 48 or 49 or 50 or 51 or 52 or 53 or 54 or 55 or 56 or 57
59. 11 and 58
60. randomized controlled trial.pt.
61. controlled clinical trial.pt.
62. randomi#ed.ab.
63. placebo$.ab.
64. drug therapy.fs.
65. randomly.ab.
66. trial.ab.
67. groups.ab.
68. 60 or 61 or 62 or 63 or 64 or 65 or 66 or 67
69. 59 and 68

# 3. EMBASE search strategy

**EMBASE (via embase.com)**

**Database: Embase Classic + Embase, 1947 to 2018 October 26.**

1. (autis* or ASD or ASDs).ti,ab.
2. PDD.ti,ab.
3. pervasive developmental disorder$.tw.
4. kanner$.tw.
5. (asperg$ not aspergill$).tw.
6. 1 or 2 or 3 or 4 or 5
7. exp child/ or adolescent/ or pediatrics/
8. (child* or schoolchild* or kid or kids or toddler* or adoles* or teen*or boy* or girl* or minors* or underag* or under age or juvenil* or youth* or kindergar* or puberty or pubescen* or prepubescen* or prepuberty* or pediatric* or paediatric* or peadiatric* or preschool* or schoolage).tw.
9. (school adj2 age*).ti,ab.
10. 7 or 8 or 9
11. 6 and 10
12. exp clinical trial/
13. exp crossover procedure/
14. exp double blind procedure/
15. exp controlled clinical trial/
16. (placebo or assign* or allocat* or volunteer* or random* or factorial* or crossover).ti,ab.
17. ((singl$ or doubl$ or trebl$ or tripl$) adj3 (blind$ or mask$)).tw.
18. 12 or 13 or 14 or 15 or 16 or 17
19. exp neuroleptic agent/
20. (anti psychotic* or antipsychotic*).mp.
21. amisulprid$.mp.
22. aripiprazol*.mp.
23. asenapin*.mp.
24. Blonanserin.mp.
25. Chlorpromazin*.mp.
26. Chlorprothixen*.mp.
27. Clotiapin*.mp.
28. Clozapin*.mp.
29. Droperidol.mp.
30. Flupentixol.mp.
31. Fluphenazin*.mp.
32. Haloperidol.mp.
33. Iloperidon*.mp.
34. Levomepromazin*.mp.
35. Loxapin*.mp.
36. Lurasidon*.mp.
37. Melperon*.mp.
38. Mesoridazin*.mp.
39. Molindon*.mp.
40. Mosapramin*.mp.
41. Olanzapin*.mp.
42. Paliperidon*.mp.
43. Periciazin*.mp.
44. Perospiron*.mp.
45. Pimozid*.mp.
46. Prochlorperazin*.mp.
47. Promazin*.mp.
48. Quetiapin*.mp.
49. Remoxiprid*.mp.
50. Risperidon*.mp.
51. Sertindol*.mp.
52. Sulpirid*.mp.
53. Thioproperazin*.mp.
54. Thioridazin*.mp.
55. Thiothixen*.mp.
56. Tiaprid*.mp.
57. Trifluoperazin*.mp.
58. Ziprasidon*.mp.
59. Zotepin*.mp.
60. Zuclopenthixol.mp.
61. Mesoridazin*.mp.
62. 19 or 20 or 21 or 22 or 23 or 24 or 25 or 26 or 27 or 28 or 29 or 30 or 31 or 32 or 33 or 34 or 35 or 36 or 37 or 38 or 39 or 40 or 41 or 42 or 43 or 44 or 45 or 46 or 47 or 48 or 49 or 50 or 51 or 52 or 53 or 54 or 55 or 56 or 57 or 58 or 59 or 60 or 61
63. 11 and 18 and 62

# 4. Web of Science search strategy

**WOS (via THOMSON REUTERS)**

Indexes=SCI-EXPANDED, SSCI, A&HCI, CPCI-S, CPCI-SSH, ESCI Timespan=All years

1. TI=(autis* or asperger* or "pervasive developmental " or (pervasive NEAR/3 child))TS=(omega 3 or omega 6)
2. TS=(anti psychotic* OR antipsychotic* OR amisulprid* OR aripiprazol* OR asenapin* OR blonanserin OR chlorpromazin* OR chlorprothixen* OR clotiapin* OR clozapin* OR droperidol OR flupentixol OR fluphenazin* OR haloperidol OR iloperidon* OR levomepromazin* OR loxapin* OR lurasidon* OR melperon* OR mesoridazin* OR molindon* OR mosapramin* OR olanzapin* OR paliperidon* OR periciazin* OR perospiron* OR pimozid* OR prochlorperazin* OR promazin* OR quetiapin* OR remoxiprid* OR risperidon* OR sertindol* OR sulpirid* OR thioproperazin* OR thioridazin* OR thiothixen* OR tiaprid* OR trifluoperazin* OR ziprasidon* OR zotepin* OR zuclopenthixol)
3. TS=(child* or schoolchild* or kid or kids or toddler* or adoles* or teen*or boy* or girl* or minors* or underag* or under age or juvenil* or youth* or kindergar* or puberty or pubescen* or prepubescen* or prepuberty* or pediatric* or paediatric* or peadiatric* or preschool* or schoolage)
4. #3 AND #2 AND #1
5. TS=(random* or control* or trial* or groups* or effectiveness or evaluation or placebo*)
6. #5 AND #4

# 5. PsycINFO search strategy

**PsycINFO (via EBSCO HOST) 1806 to present**

1. (autis* or ASD or ASDs).ti,ab.
2. PDD.ti,ab.
3. pervasive developmental disorder$.tw.
4. kanner$.tw.
5. (asperg$ not aspergill$).tw.
6. 1 or 2 or 3 or 4 or 5
7. exp child/ or adolescent/ or pediatrics/
8. (child* or schoolchild* or kid or kids or toddler* or adoles* or teen*or boy* or girl* or minors* or underag* or under age or juvenil* or youth* or kindergar* or puberty or pubescen* or prepubescen* or prepuberty* or pediatric* or paediatric* or peadiatric* or preschool* or schoolage).tw.
9. (school adj2 age*).ti,ab.
10. 7 or 8 or 9
11. 6 and 10
12. exp clinical trial/
13. (placebo or assign* or allocat* or volunteer* or random* or factorial* or crossover).ti,ab.
14. ((singl$ or doubl$ or trebl$ or tripl$) adj3 (blind$ or mask$)).tw.
15. (anti psychotic* or antipsychotic*).mp.
16. amisulprid$.mp.
17. aripiprazol*.mp.
18. asenapin*.mp.
19. Blonanserin.mp.
20. Chlorpromazin*.mp.
21. Chlorprothixen*.mp.
22. Clotiapin*.mp.
23. Clozapin*.mp.
24. Droperidol.mp.
25. Flupentixol.mp.
26. Fluphenazin*.mp.
27. Haloperidol.mp.
28. Iloperidon*.mp.
29. Levomepromazin*.mp.
30. Loxapin*.mp.
31. Lurasidon*.mp.
32. Melperon*.mp.
33. Mesoridazin*.mp.
34. Molindon*.mp.
35. Mosapramin*.mp.
36. Olanzapin*.mp.
37. Paliperidon*.mp.
38. Periciazin*.mp.
39. Perospiron*.mp.
40. Pimozid*.mp.
41. Prochlorperazin*.mp.
42. Promazin*.mp.
43. Quetiapin*.mp.
44. Remoxiprid*.mp.
45. Risperidon*.mp.
46. Sertindol*.mp.
47. Sulpirid*.mp.
48. Thioproperazin*.mp.
49. Thioridazin*.mp.
50. Thiothixen*.mp.
51. Tiaprid*.mp.
52. Trifluoperazin*.mp.
53. Ziprasidon*.mp.
54. Zotepin*.mp.
55. Zuclopenthixol.mp.
56. Mesoridazin*.mp.
57. 12 or 13 or 14
58. 15 or 16 or 17 or 18 or 19 or 20 or 21 or 22 or 23 or 24 or 25 or 26 or 27 or 28 or 29 or 30 or 31 or 32 or 33 or 34 or 35 or 36 or 37 or 38 or 39 or 40 or 41 or 42 or 43 or 44 or 45 or 46 or 47 or 48 or 49 or 50 or 51 or 52 or 53 or 54 or 55 or 56
59. 11 and 57 and 58

We searched for ongoing clinical trials and unpublished trials via Internet searches on the following web-sites:

- ClinicalTrials.gov ([www.clinicaltrials.gov](http://www.clinicaltrials.gov/));
- World Health Organization (WHO) International Clinical Trials Registry Platform (ICTRP) ([apps.who.int/trialsearch/](http://apps.who.int/trialsearch/)).

# Numbers of citations by each database

| **Databases, trial registers and other sources** | **Citations** |
| --- | --- |
| **Databases:** |  |
| PubMed | 696 |
| Central | 215 |
| Web of Science | 418 |
| Embase | 492 |
| PsycINFO | 166 |
| **Total (databases)** | **1987** |
| Duplicate records removed | 779 |
| **Total (databases) after duplicates removed** | **1208** |
|  |  |
| **Trial registers:** |  |
| ClinicalTrials.gov | 52 |
| World Health Organization (WHO) International Clinical Trials Registry Platform (ICTRP) ([apps.who.int/trialsearch/](http://apps.who.int/trialsearch/)). | 0 |
| **Total (trial registers)** | **52** |
|  |  |
| **Other sources:** | **0** |
|  |  |
| **Total citations:** | **1260** |
