# Supplementary material for: Impact of antipsychotics in children and adolescents with autism spectrum disorder: a systematic review and meta-analysis
Source: Health Qual Life Outcomes. 2021 Jan 25;19:33. doi: 10.1186/s12955-021-01669-0 (PMC7831175; doi:10.1186/s12955-021-01669-0)
Supplement: Supplementary file 4 — Additional file 4: Risk of Bias Summary.. [file 12955_2021_1669_MOESM4_ESM.docx]

**Additional file 4. Risk of Bias Summary.**

**These are review authors' judgments about each risk of bias item for each included study.**

| **Study** | **Random sequence generation**  **(selection bias)** | **Allocation concealment (selection bias)** | **Blinding of participants and personnel (performance bias)** | **Blinding of outcome assessment**  **(detection bias)** | **Incomplete outcome data**  **(attrition bias)** | **Selective reporting (reporting bias)** |
| --- | --- | --- | --- | --- | --- | --- |
| Anderson, 1984 ^1^ | 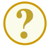 | 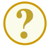 | 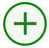 | 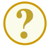 | 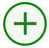 | 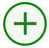 |
| Anderson, 1989 ^2^ | 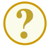 | 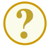 | 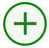 | 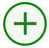 | 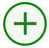 | 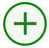 |
| Campbell, 1978 ^3^ | 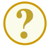 | 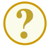 | 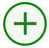 | 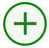 | 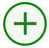 | 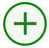 |
| Cohen, 1980 ^4^ | 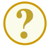 | 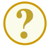 | 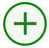 | 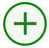 | 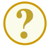 | 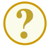 |
| Findling, 2014 ^5^ | 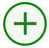 | 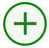 | 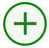 | 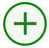 | **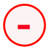** | 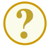 |
| Hellings, 2006 ^6^ | 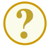 | 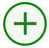 | 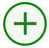 | 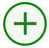 | 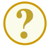 | 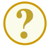 |
| Hollander, 2006 ^7^ | 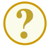 | 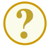 | 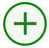 | 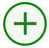 | 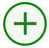 | 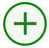 |
| Ichikawa, 2017 ^8^ | 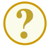 | 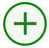 | 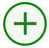 | 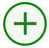 | 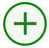 | 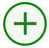 |
| Kent, 2013 ^9^ | 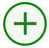 | 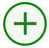 | 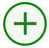 | 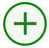 | 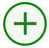 | 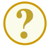 |
| Loebel, 2016 ^10^ | 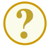 | 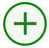 | 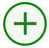 | 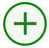 | 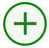 | 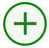 |
| Luby, 2006 ^11^ | 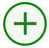 | **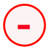** | 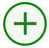 | 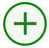 | 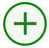 | **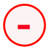** |
| Marcus, 2009 ^12^ | 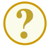 | 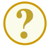 | 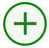 | 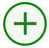 | 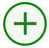 | 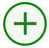 |
| McCraken, 2002 ^13^ | 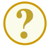 | 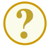 | 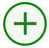 | 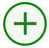 | **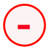** | 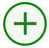 |
| Nagaraj, 2006 ^14^ | 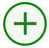 | 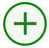 | 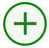 | 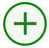 | 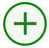 | 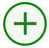 |
| NCT00870727 ^15^ | 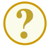 | 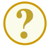 | 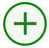 | 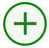 | 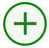 | 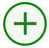 |
| NCT01624675 ^16^ | 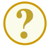 | 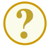 | 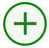 | 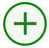 | **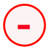** | 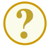 |
| Owen, 2009 ^17^ | 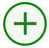 | 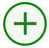 | 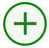 | 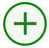 |  |  |
| Remington, 2001 ^18^ |  |  |  |  |  |  |
| RUPP, 2005 ^19^ |  |  |  |  |  |  |
| Shea, 2004 ^20^ |  |  |  |  |  |  |
| Troost, 2005 ^21^ |  |  |  |  |  |  |

Abbreviations: = Low risk of bias; = Unclear risk of bias;  = High risk of bias.

**References**

1. Anderson LT, Campbell M, Grega DM, Perry R, Small AM, Green WH. Haloperidol in the treatment of infantile autism: effects on learning and behavioral symptoms. Am J Psychiatry. 1984 Oct;141(10):1195-202. PubMed PMID: 6385731.
2. Anderson LT, Campbell M, Adams P, et al. The effects of haloperidol on discrimination learning and behavioral symptoms in autistic children. J Autism Dev Disord. 1989 Jun;19(2):227-39. PMID: 2663834.
3. Campbell M, Anderson LT, Meier M, Cohen IL, Small AM, Samit C, Sachar EJ. A comparison of haloperidol and behavior therapy and their interaction in autistic children. J Am Acad Child Psychiatry. 1978 Autumn;17(4):640-55. PubMed PMID: 370186.
4. Cohen IL, Campbell M, Posner D, Small AM, Triebel D, Anderson LT. Behavioral effects of haloperidol in young autistic children. An objective analysis using a within-subjects reversal design. J Am Acad Child Psychiatry. 1980 Autumn;19(4):665-77. PubMed PMID: 7204797.
5. Findling RL, Mankoski R, Timko K, Lears K, McCartney T, McQuade RD, Eudicone JM, Amatniek J, Marcus RN, Sheehan JJ. A randomized controlled trial investigating the safety and efficacy of aripiprazole in the long-term maintenance treatment of pediatric patients with irritability associated with autistic disorder. J Clin Psychiatry. 2014 Jan;75(1):22-30. doi:10.4088/JCP.13m08500. PubMed PMID: 24502859.
6. Hellings JA, Zarcone JR, Reese RM, Valdovinos MG, Marquis JG, Fleming KK, Schroeder SR. A crossover study of risperidone in children, adolescents and adults with mental retardation. J Autism Dev Disord. 2006 Apr;36(3):401-11. PubMed PMID: 16596465.
7. Hollander E, Wasserman S, Swanson EN, et al. A double-blind placebo-controlled pilot study of olanzapine in childhood/adolescent pervasive developmental disorder. J Child Adolesc Psychopharmacol. 2006;16(5):541-8. doi:10.1089/cap.2006.16.541. PMID: 17069543.
8. Ichikawa H, Mikami K, Okada T, Yamashita Y, Ishizaki Y, Tomoda A, Ono H, Usuki C, Tadori Y. Aripiprazole in the Treatment of Irritability in Children and Adolescents with Autism Spectrum Disorder in Japan: A Randomized, Double-blind, Placebo-controlled Study. Child Psychiatry Hum Dev. 2017 Oct;48(5):796-806. doi: 10.1007/s10578-016-0704-x. PubMed PMID: 28004215; PubMed Central PMCID: PMC5617873.
9. Kent JM, Kushner S, Ning X, et al. Risperidone dosing in children and adolescents with autistic disorder: a double-blind, placebo-controlled study. J Autism Dev 197 Disord. 2013 Aug;43(8):1773-83. doi: 10.1007/s10803-012-1723-5. PMID: 23212807.
10. Loebel A, Brams M, Goldman RS, et al. Lurasidone for the treatment of irritability with autistic disorder. J Autism Dev Disord. 2016;46:1153-63.
11. Luby J, Mrakotsky C, Stalets MM, et al. Risperidone in preschool children with autistic spectrum disorders: an investigation of safety and efficacy. J Child Adoles Psychopharmacol. 2006;16(5):575-87. doi:10.1089/cap.2006.16.575. PMID: 17069546.
12. Marcus RN, Owen R, Kamen L, Manos G, McQuade RD, Carson WH, Aman MG. A placebo-controlled, fixed-dose study of aripiprazole in children and adolescents with irritability associated with autistic disorder. J Am Acad Child Adolesc Psychiatry. 2009 Nov;48(11):1110-9. doi: 10.1097/CHI.0b013e3181b76658. PubMed PMID: 19797985.
13. McCracken JT, McGough J, Shah B, et al. Risperidone in children with autism and serious behavioral problems. N Engl J Med. 2002;347(5):314-21. doi: 10.1056/NEJMoa013171 PMID: 12151468.
14. Nagaraj R, Singhi P, Malhi P. Risperidone in children with autism: randomized, placebo-controlled, double-blind study. J Child Neurol. 2006;21(6):450-5. doi: 10.1177/08830738060210060801. PMID: 16948927.
15. NCT00870727. Study of Aripiprazole in the Treatment of Pervasive Developmental Disorders. First posted: 27^th^ Mar 2009. Accessed: 18^th^ Feb 2019.
16. NCT01624675. A Study to Evaluate the Efficacy and Safety of Risperidone (R064766) in Children and Adolescents With Irritability Associated With Autistic Disorder. First posted: 21^st^ Jun 2012. Accessed: 18^th^ Feb 2019.
17. Owen R, Sikich L, Marcus RN, Corey-Lisle P, Manos G, McQuade RD, Carson WH, Findling RL. Aripiprazole in the treatment of irritability in children and adolescents with autistic disorder. Pediatrics. 2009 Dec;124(6):1533-40. doi: 10.1542/peds.2008-3782. PubMed PMID: 19948625.
18. Remington G, Sloman L, Konstantareas M, et al. Clomipramine versus haloperidol in the treatment of autistic disorder: a double-blind, placebo-controlled, crossover study. J Clin Psychopharmacol. 2001 Aug;21(4):440-4. PMID: 11476129.
19. Research Units on Pediatric Psychopharmacology (RUPP). Risperidone treatment of autistic disorder: longer-term benefits and blinded discontinuation after 6 months. Am J Psychiatry. 2005;162(7):1361-9.
20. Shea S, Turgay A, Carroll A, et al. Risperidone in the treatment of disruptive behavioral symptoms in children with autistic and other pervasive developmental disorders. Pediatrics. 2004;114(5):e634-e41. PMID: 15492353.
21. Troost PW, Lahuis BE, Steenhuis MP, et al. Long-term effects of risperidone in children with autism spectrum disorders: a placebo discontinuation study. J Am Acad Child Adolesc Psychiatry. 2005;44(11):1137-44. doi:10.1097/01.chi.0000177055.11229.76 PMID: 16239862.
