# Supplementary material for: Impact of antipsychotics in children and adolescents with autism spectrum disorder: a systematic review and meta-analysis
Source: Health Qual Life Outcomes. 2021 Jan 25;19:33. doi: 10.1186/s12955-021-01669-0 (PMC7831175; doi:10.1186/s12955-021-01669-0)

Additional file 5: Forest plots of comparisons between antipsychotics (D2 blockers) and Placebo

[RESTRICTED AND REPETITIVE INTERESTS AND BEHAVIORS 2](#_Toc26793336)

[HYPERACTIVITY, INATTENTION, OPPOSITIVENESS, DISRUPTIVE BEHAVIOR. 3](#_Toc26793337)

[SELF-HARM 3](#_Toc26793338)

[SOCIAL COMMUNICATION, SOCIAL INTERACTION 4](#_Toc26793339)

[EMOTIONAL DYSREGULATION/IRRITABILITY 5](#_Toc26793340)

[ANXIETY 6](#_Toc26793341)

[GLOBAL FUNCTIONING, GLOBAL IMPROVEMENT 7](#_Toc26793342)

[OBSESSIONS, COMPULSIONS 8](#_Toc26793343)

[SEVERE ADVERSE EVENTS 9](#_Toc26793344)

[ADVERSE EVENTS 10](#_Toc26793345)

[DROPOUT DUE TO ANY CAUSE 11](#_Toc26793346)

[DROPOUT DUE TO ADVERSE EVENTS 12](#_Toc26793347)

## RESTRICTED AND REPETITIVE INTERESTS AND BEHAVIORS


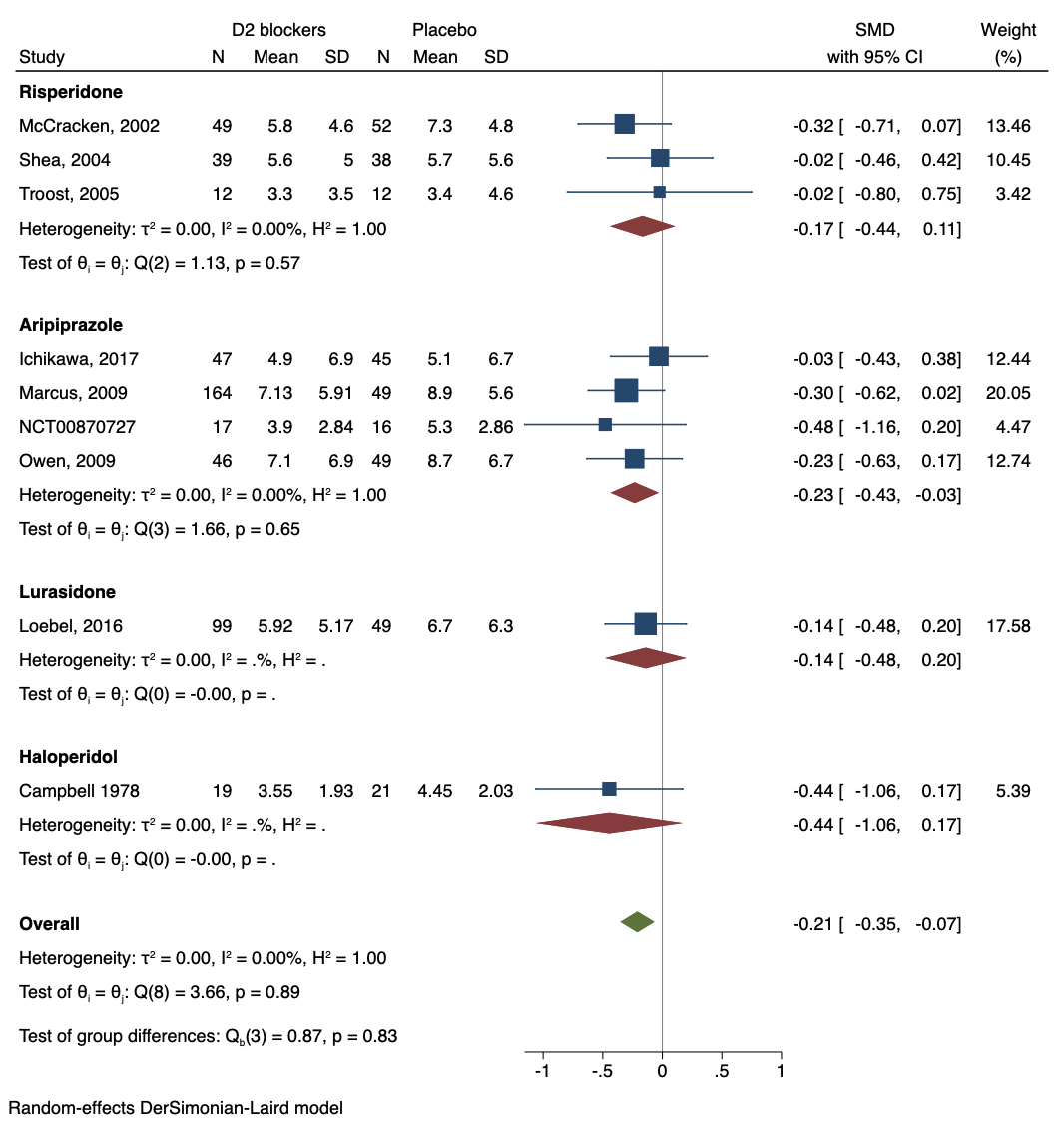


## HYPERACTIVITY, INATTENTION, OPPOSITIVENESS, DISRUPTIVE BEHAVIOR.


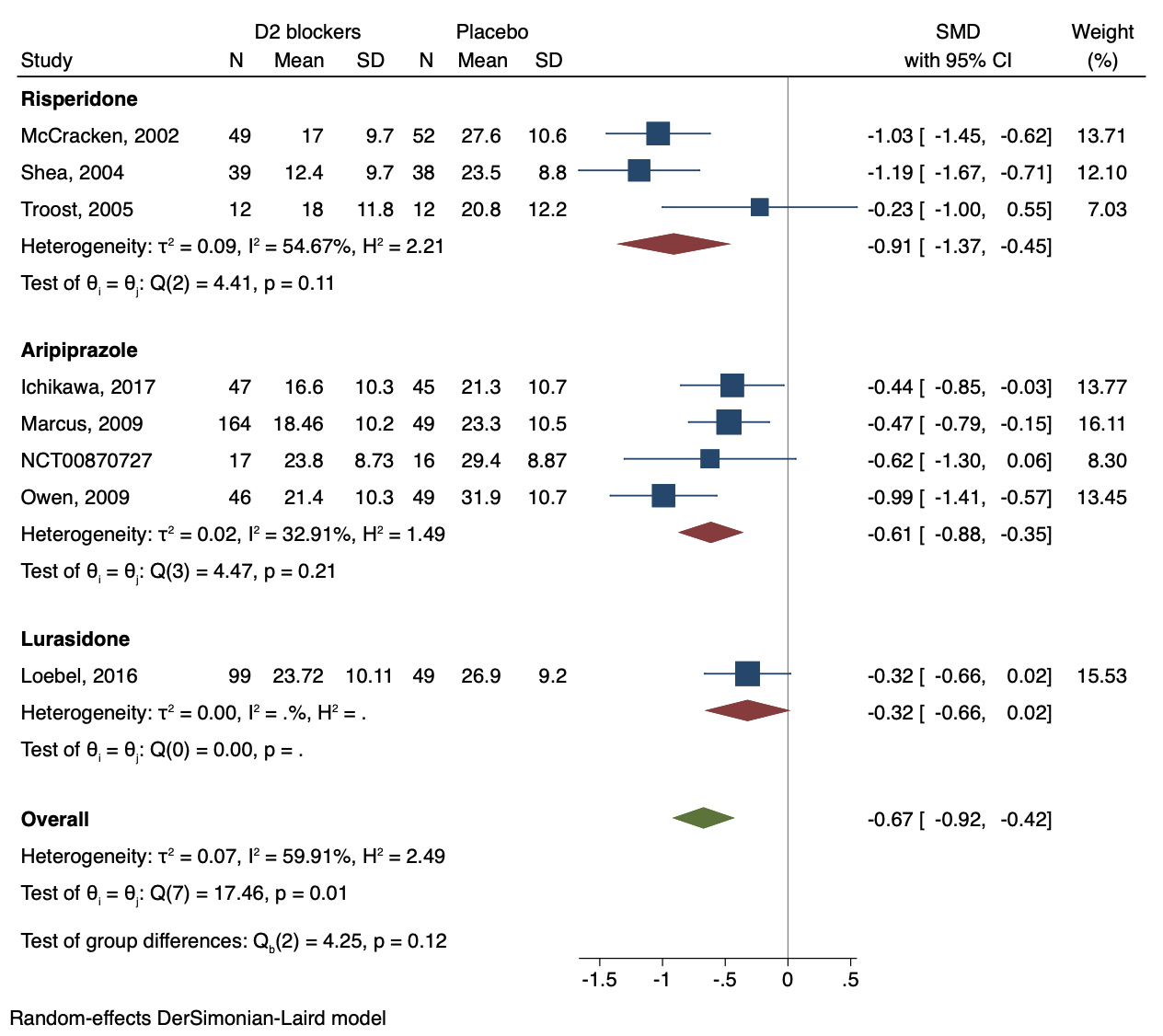


## SELF-HARM


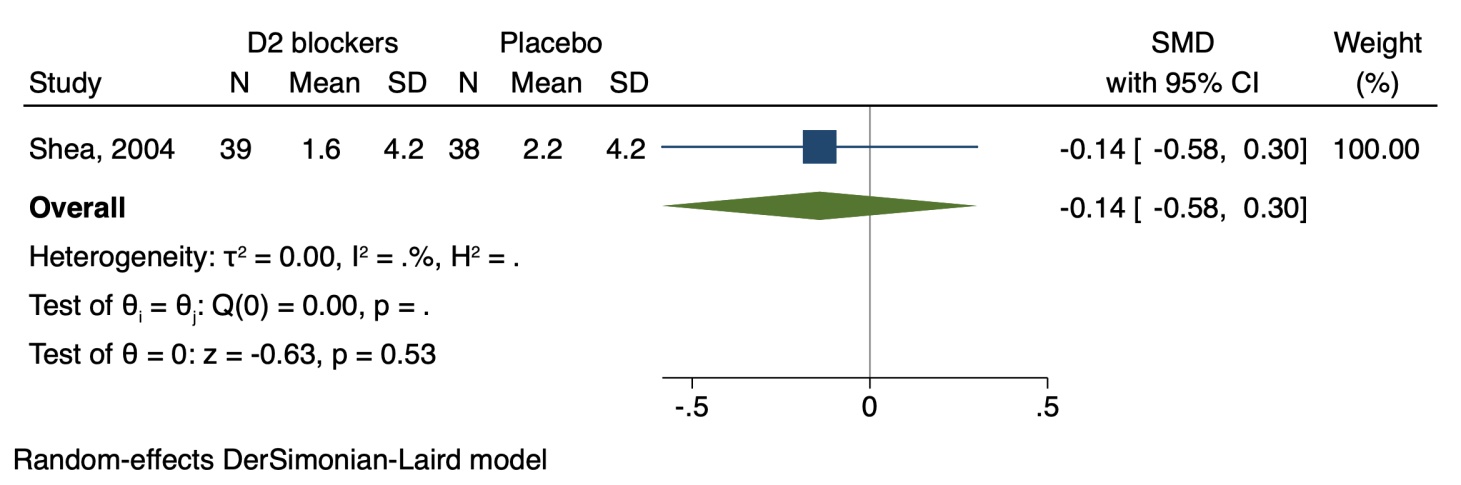


## SOCIAL COMMUNICATION, SOCIAL INTERACTION


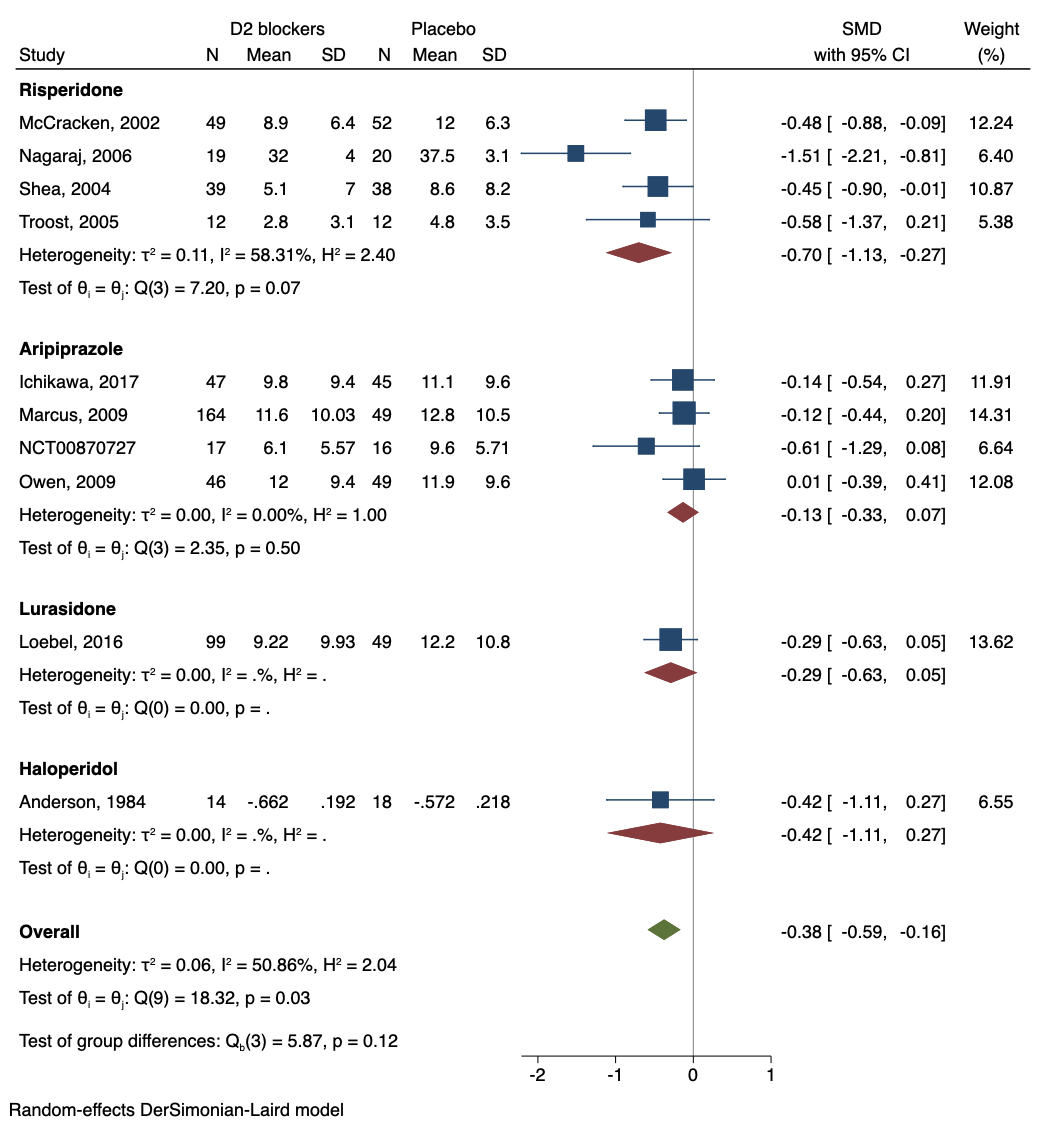


## EMOTIONAL DYSREGULATION/IRRITABILITY


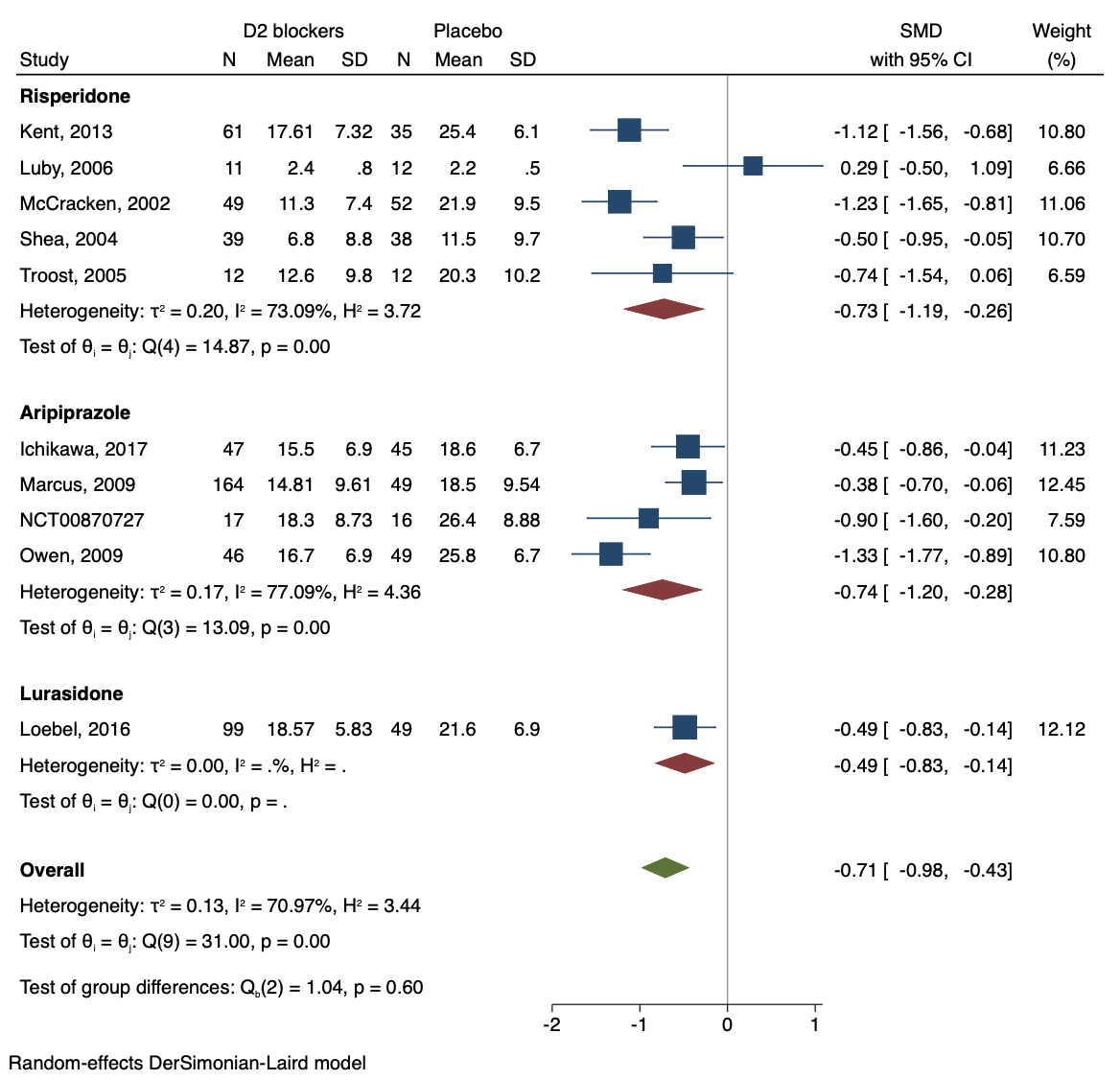


## ANXIETY


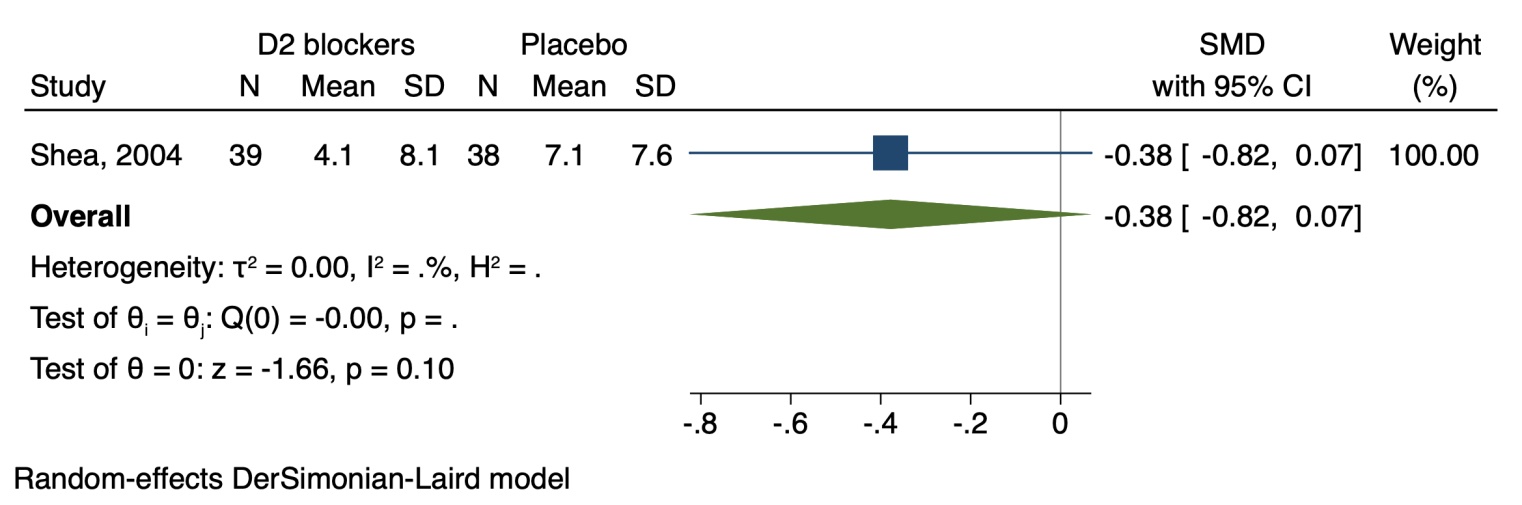


## GLOBAL FUNCTIONING, GLOBAL IMPROVEMENT


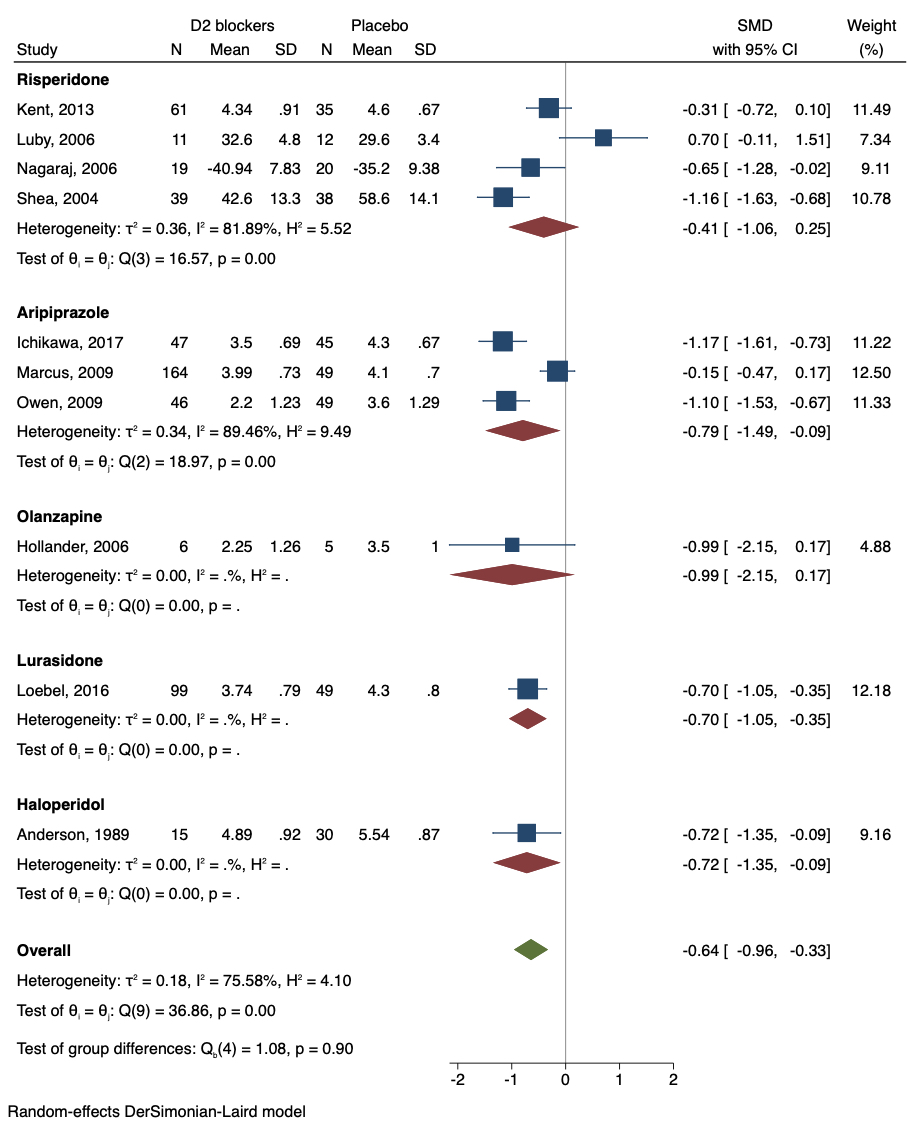


## OBSESSIONS, COMPULSIONS


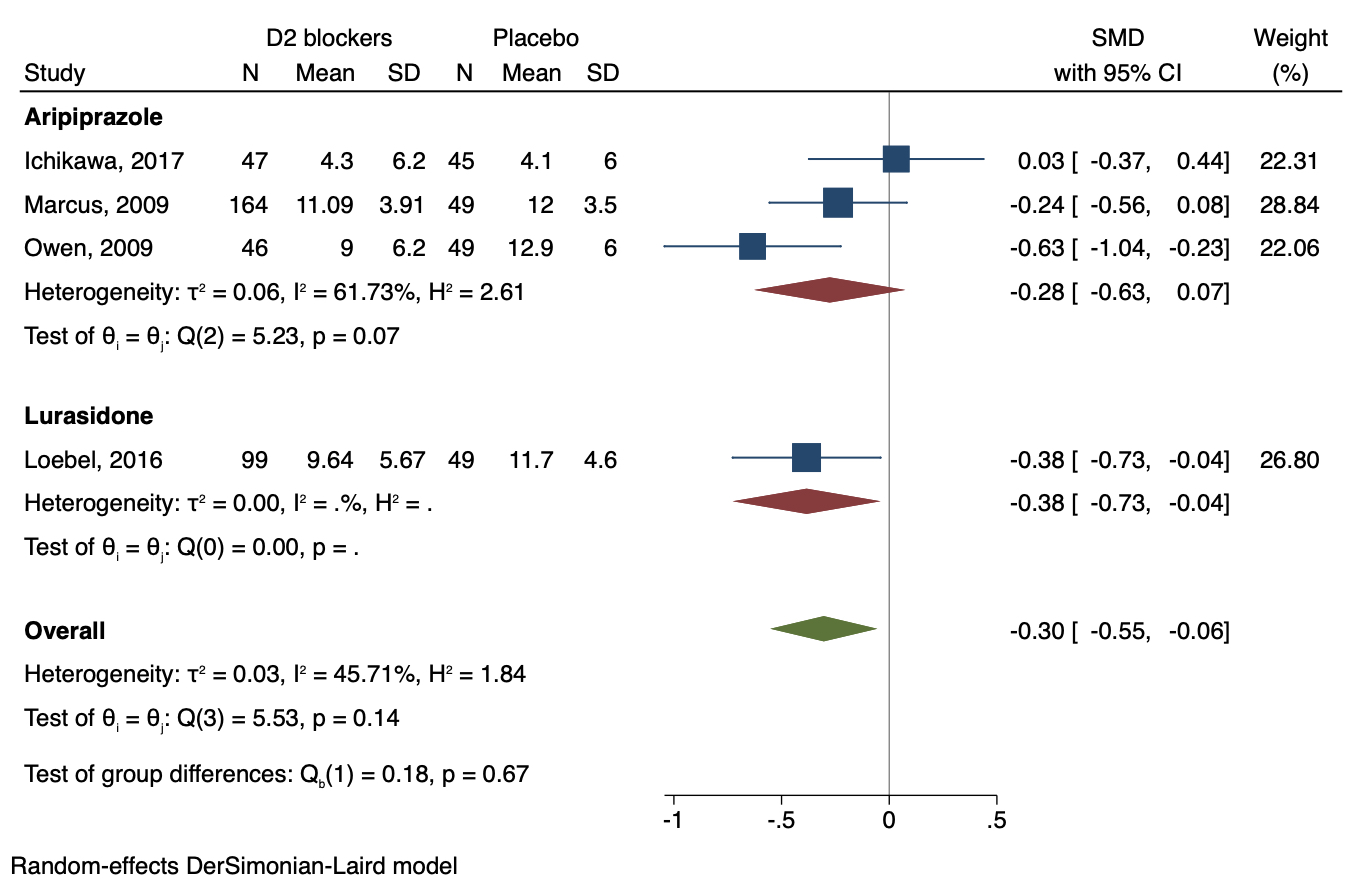


## SEVERE ADVERSE EVENTS


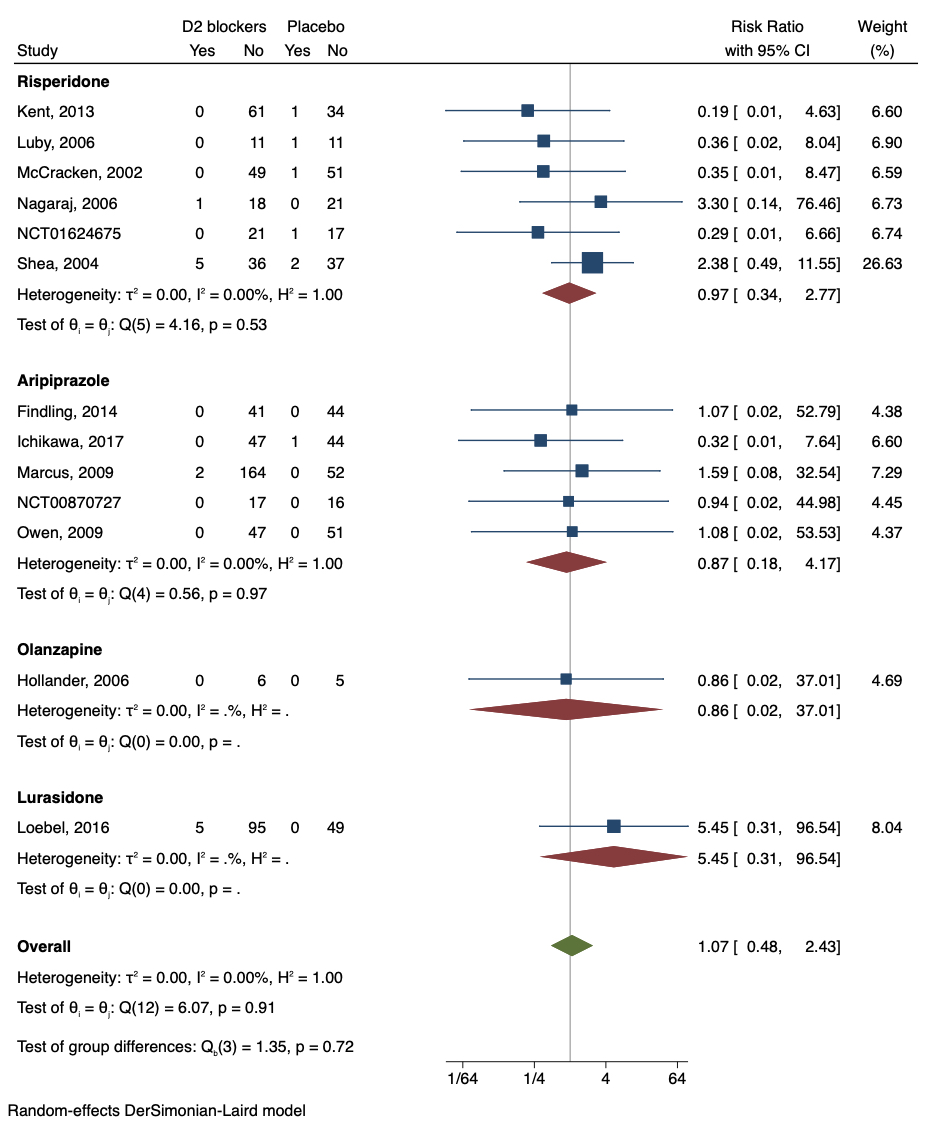


## ADVERSE EVENTS


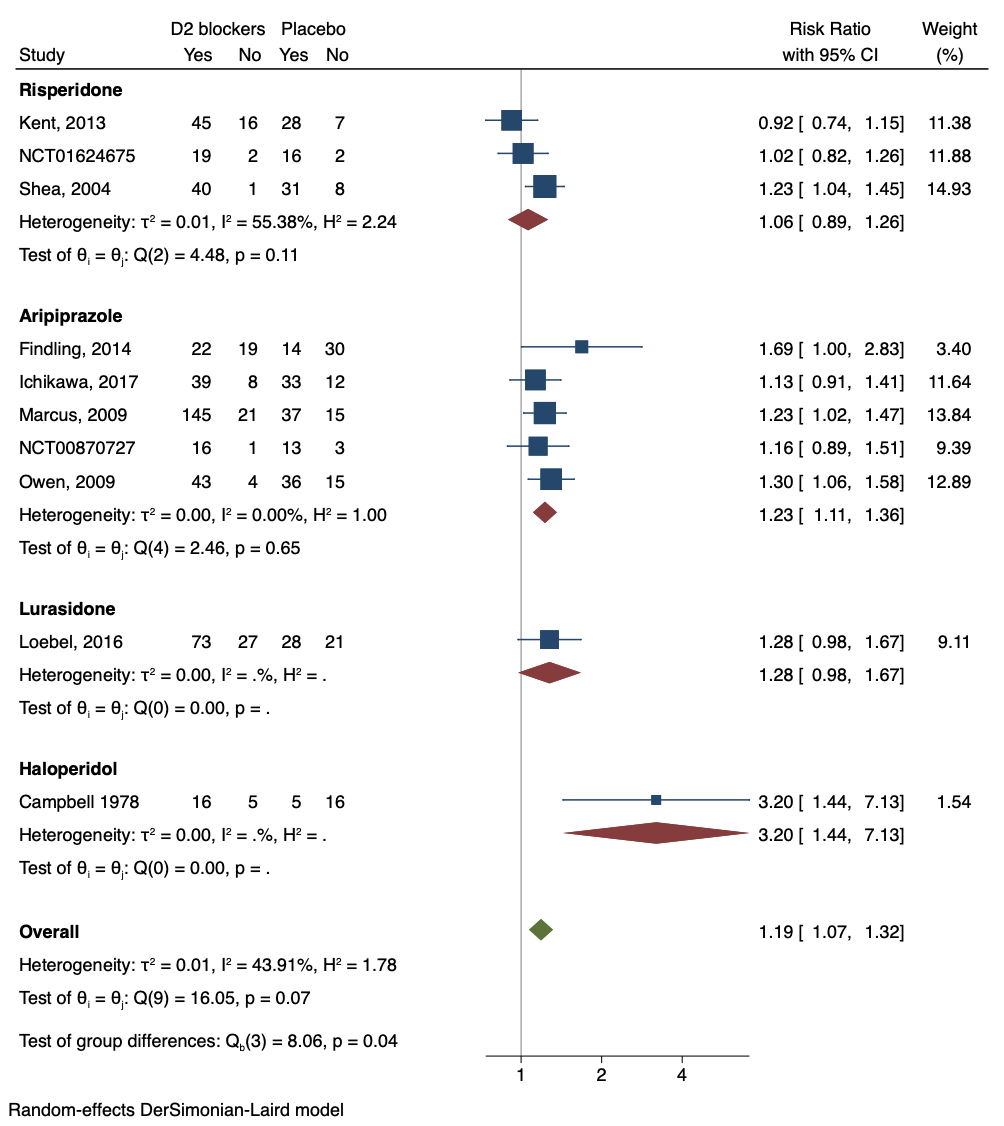


## DROPOUT DUE TO ANY CAUSE


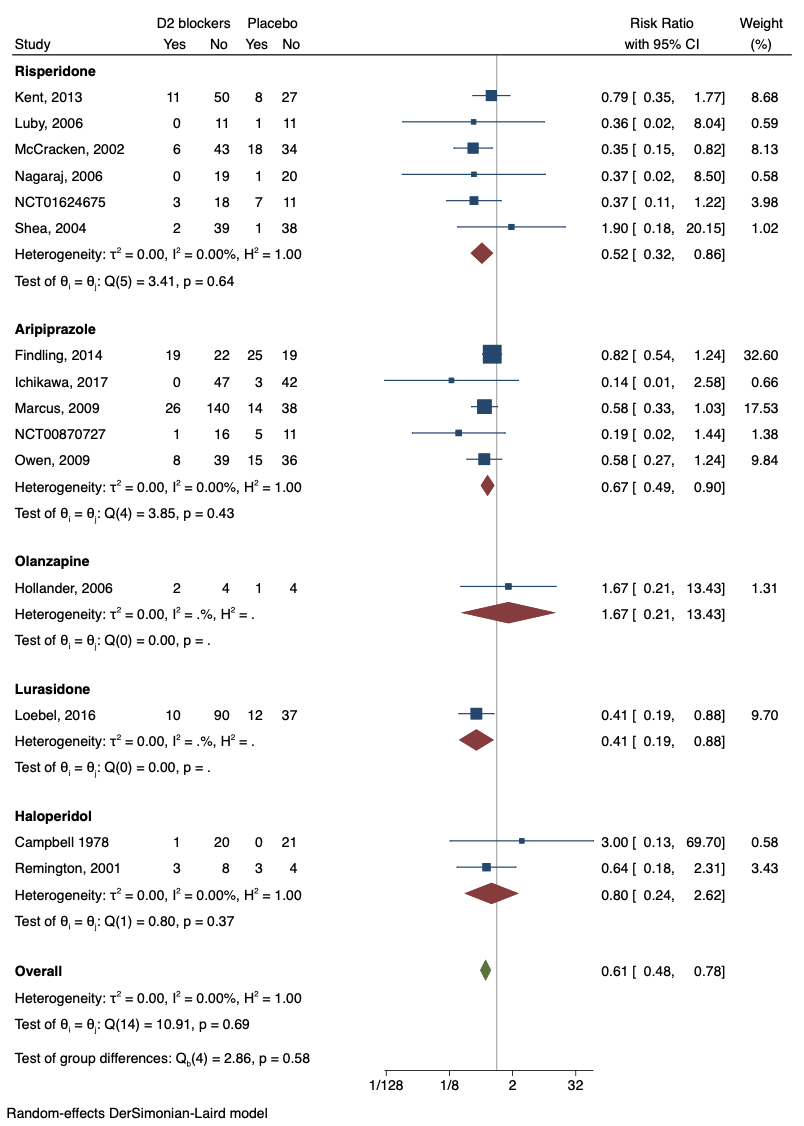


## DROPOUT DUE TO ADVERSE EVENTS


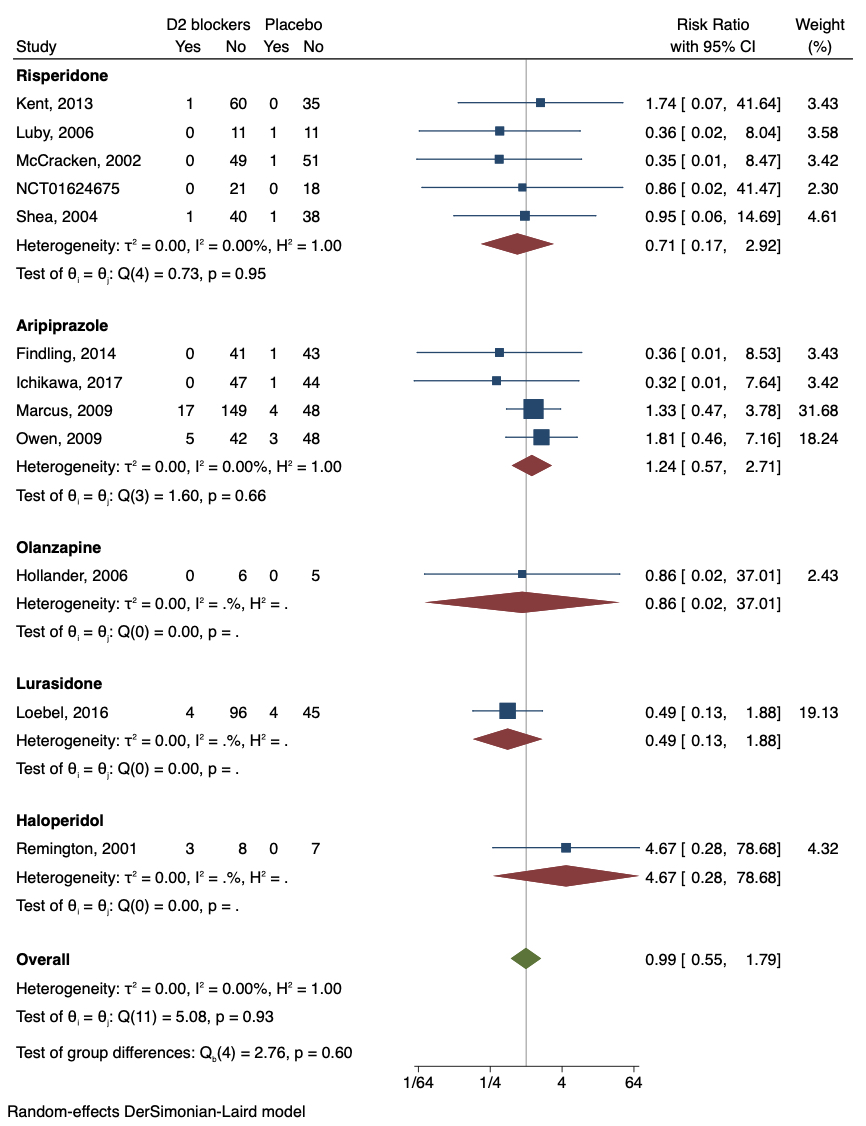

Supplement: Supplementary file 5 — Additional file 5: Forest plots for comparisons between antipsychotics (D2 blockers) and Placebo. [file 12955_2021_1669_MOESM5_ESM.docx]
