# Supplementary material for: Impact of antipsychotics in children and adolescents with autism spectrum disorder: a systematic review and meta-analysis
Source: Health Qual Life Outcomes. 2021 Jan 25;19:33. doi: 10.1186/s12955-021-01669-0 (PMC7831175; doi:10.1186/s12955-021-01669-0)

Additional file 7. Funnel Plots for outcome with estimates for at least 8 studies.

[RESTRICTED AND REPETITIVE INTERESTS AND BEHAVIORS 2](#_Toc26796813)

[HYPERACTIVITY, INATTENTION, OPPOSITIVENESS, DISRUPTIVE BEHAVIOR. 3](#_Toc26796814)

[SOCIAL COMMUNICATION, SOCIAL INTERACTION 4](#_Toc26796815)

[EMOTIONAL DYSREGULATION/IRRITABILITY 5](#_Toc26796816)

[GLOBAL FUNCTIONING, GLOBAL IMPROVEMENT 6](#_Toc26796817)

[SEVERE ADVERSE EVENTS 7](#_Toc26796818)

[ADVERSE EVENTS 8](#_Toc26796819)

[DROPOUT DUE TO ANY CAUSE 9](#_Toc26796820)

[DROPOUT DUE TO ADVERSE EVENTS 10](#_Toc26796821)

## RESTRICTED AND REPETITIVE INTERESTS AND BEHAVIORS


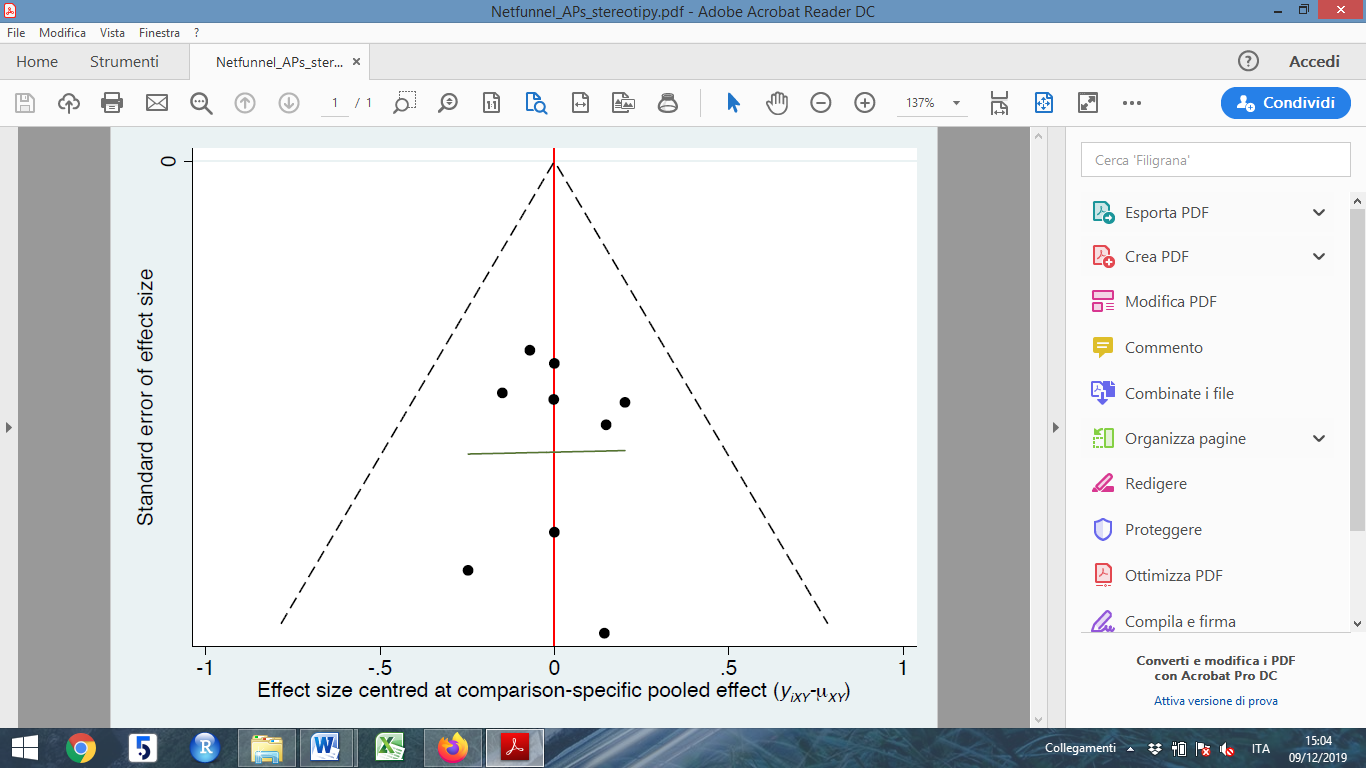


## HYPERACTIVITY, INATTENTION, OPPOSITIVENESS, DISRUPTIVE BEHAVIOR.


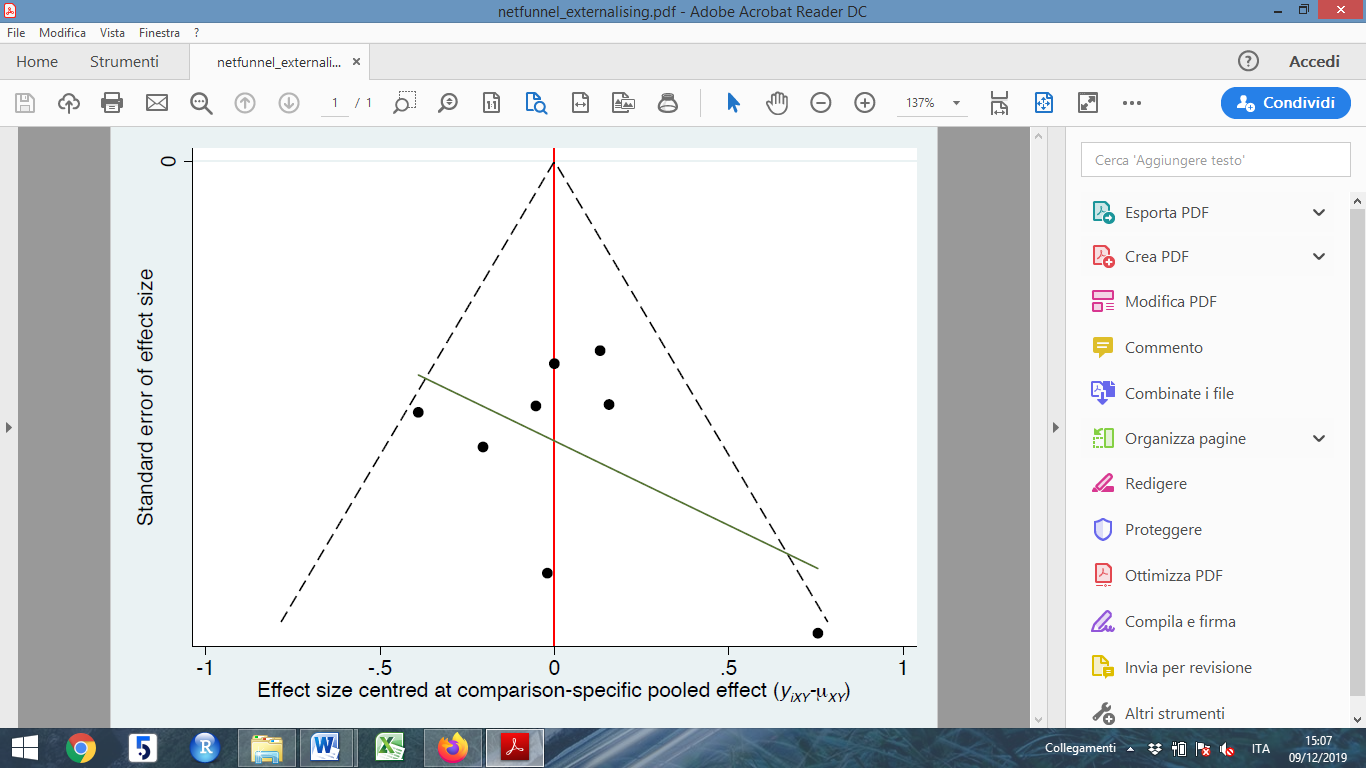


## SOCIAL COMMUNICATION, SOCIAL INTERACTION


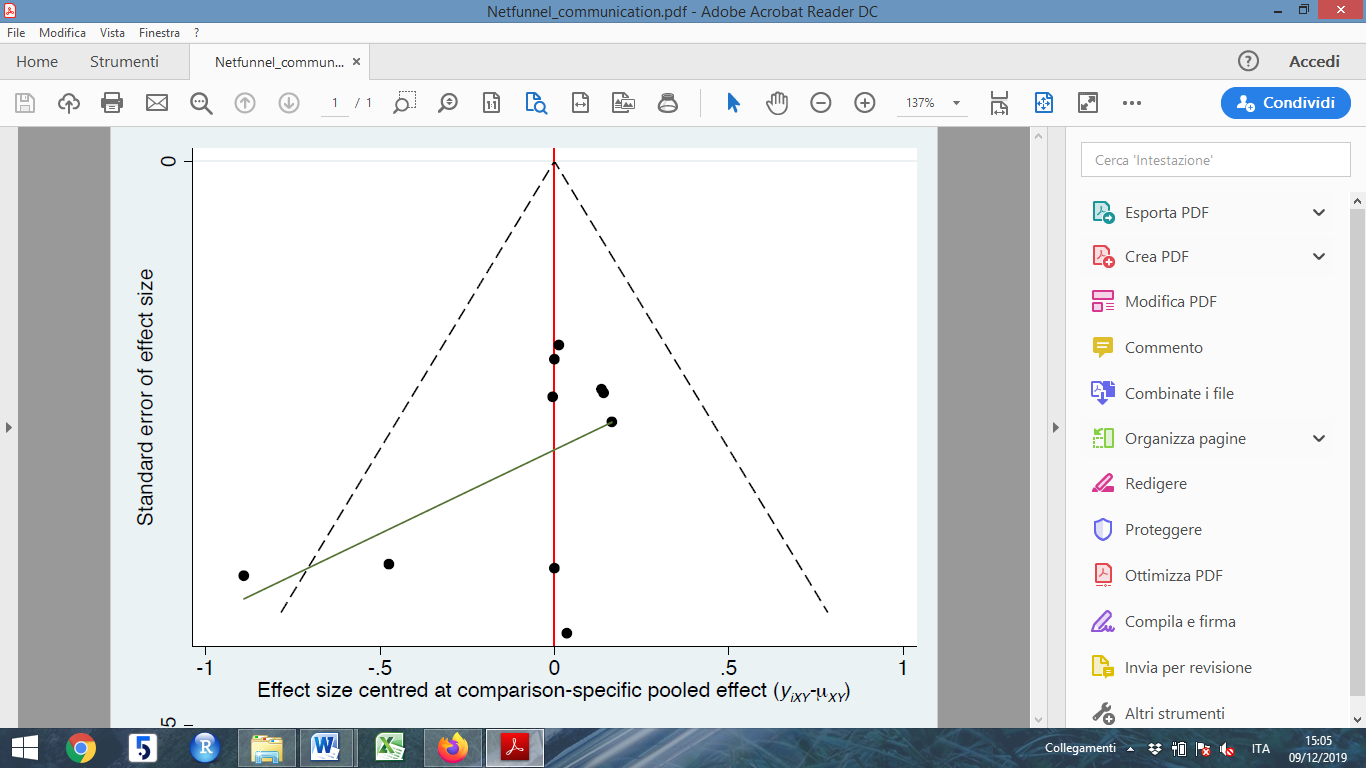


## EMOTIONAL DYSREGULATION/IRRITABILITY


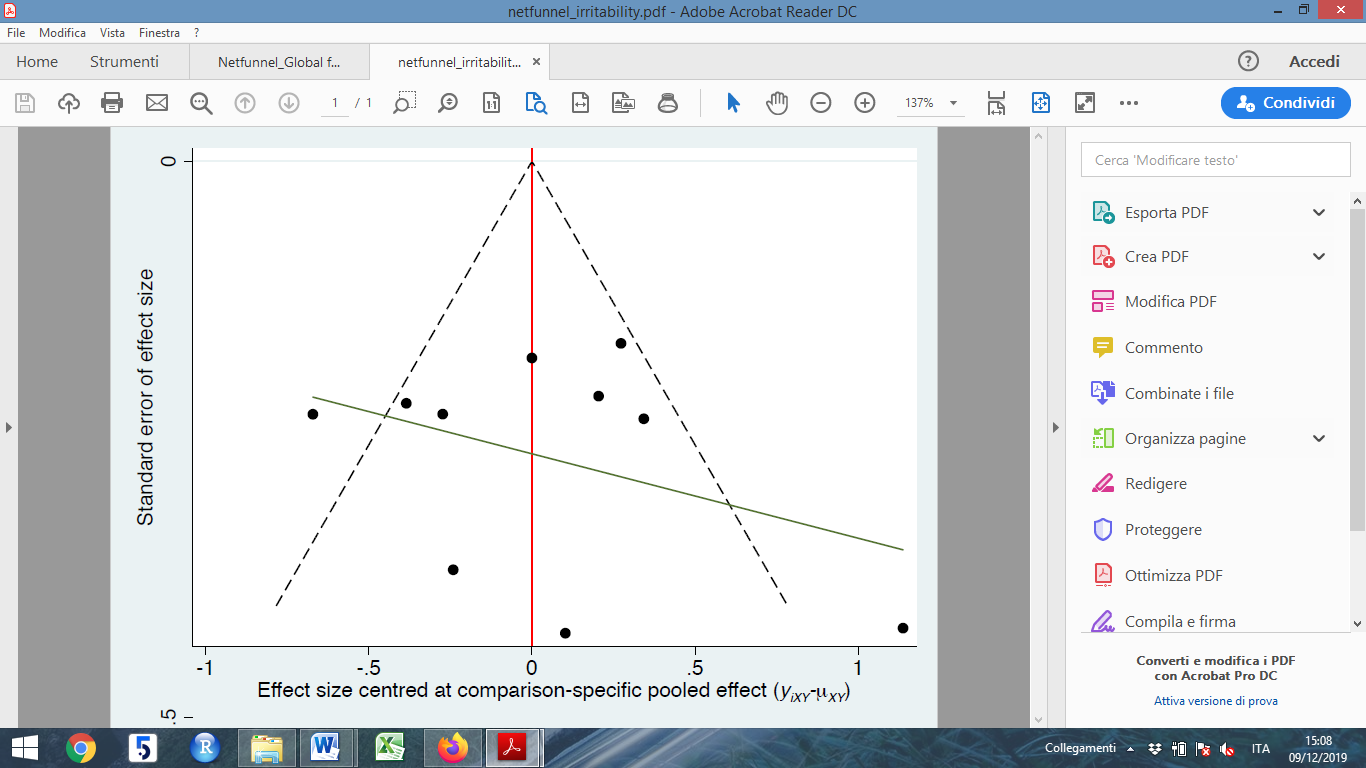


## GLOBAL FUNCTIONING, GLOBAL IMPROVEMENT


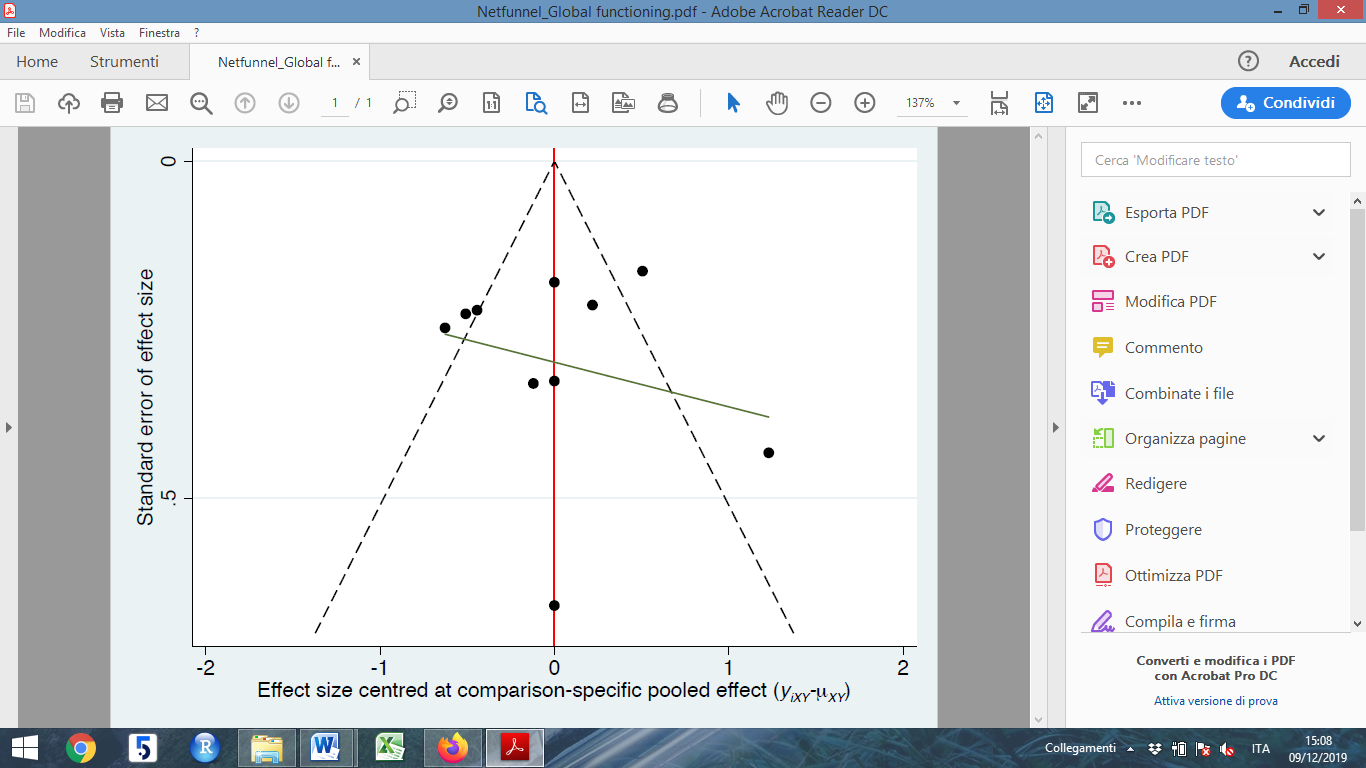


## SEVERE ADVERSE EVENTS


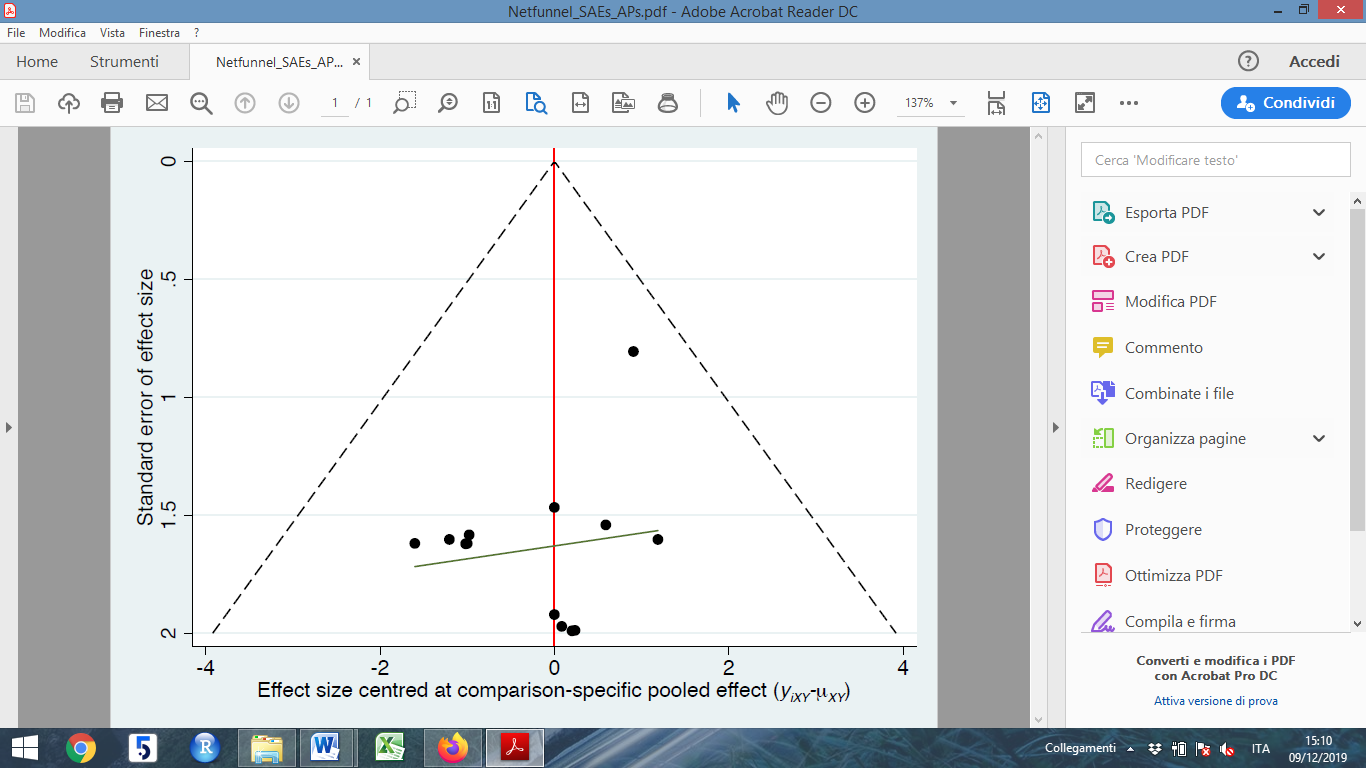


## ADVERSE EVENTS


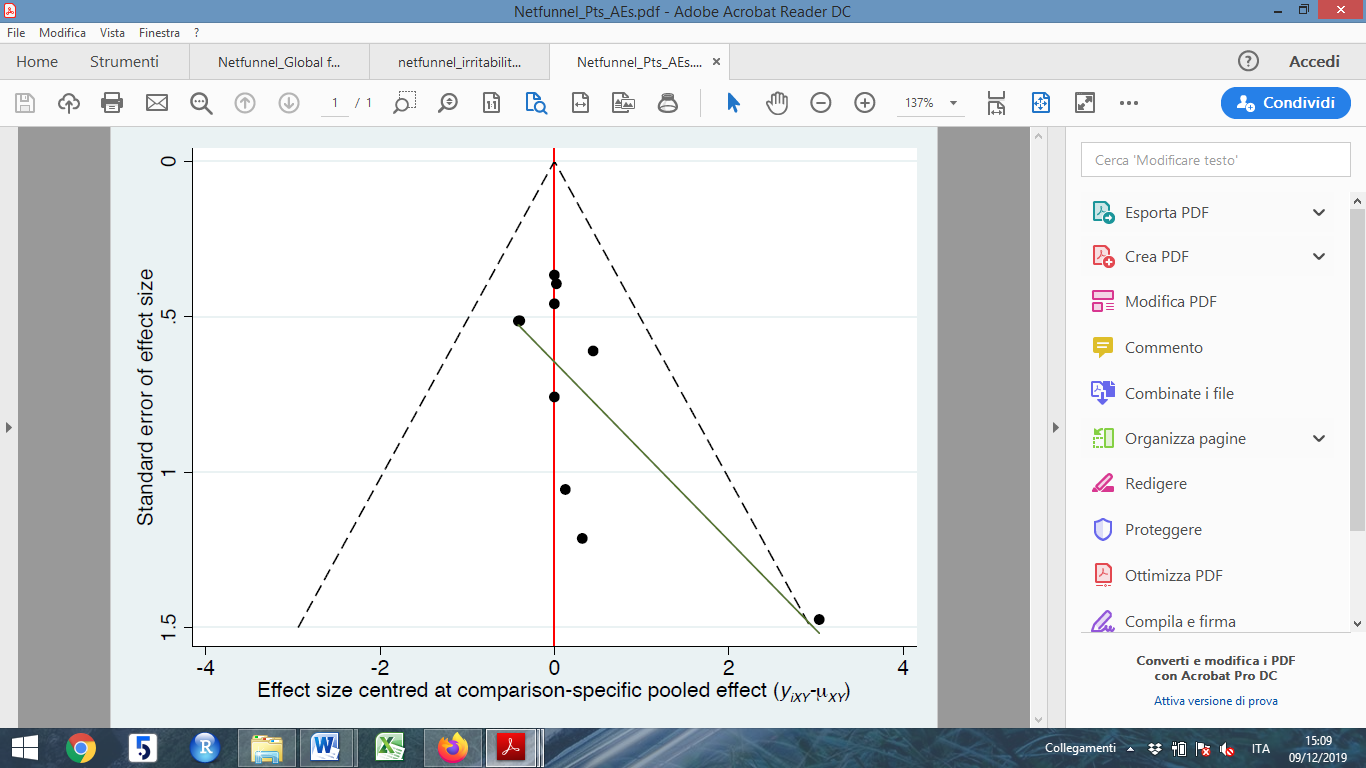


## DROPOUT DUE TO ANY CAUSE


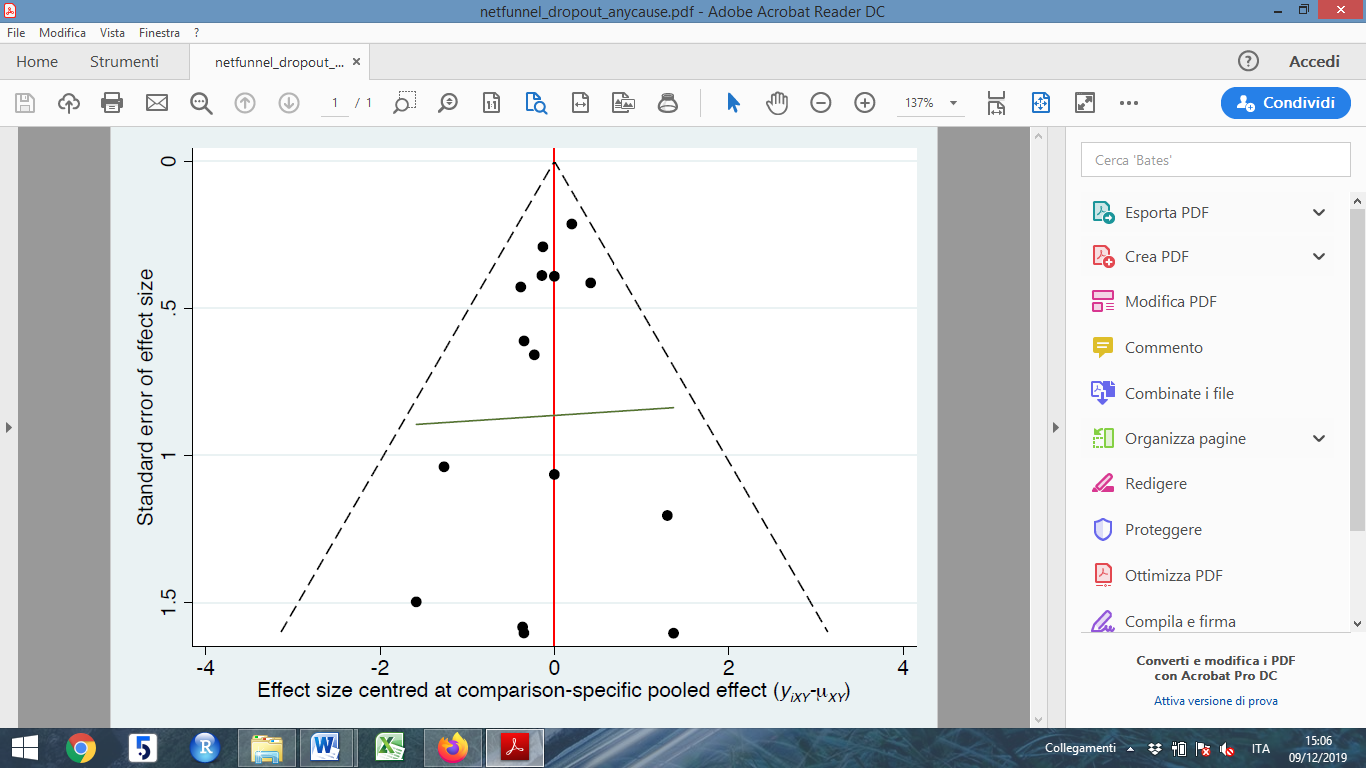


## DROPOUT DUE TO ADVERSE EVENTS


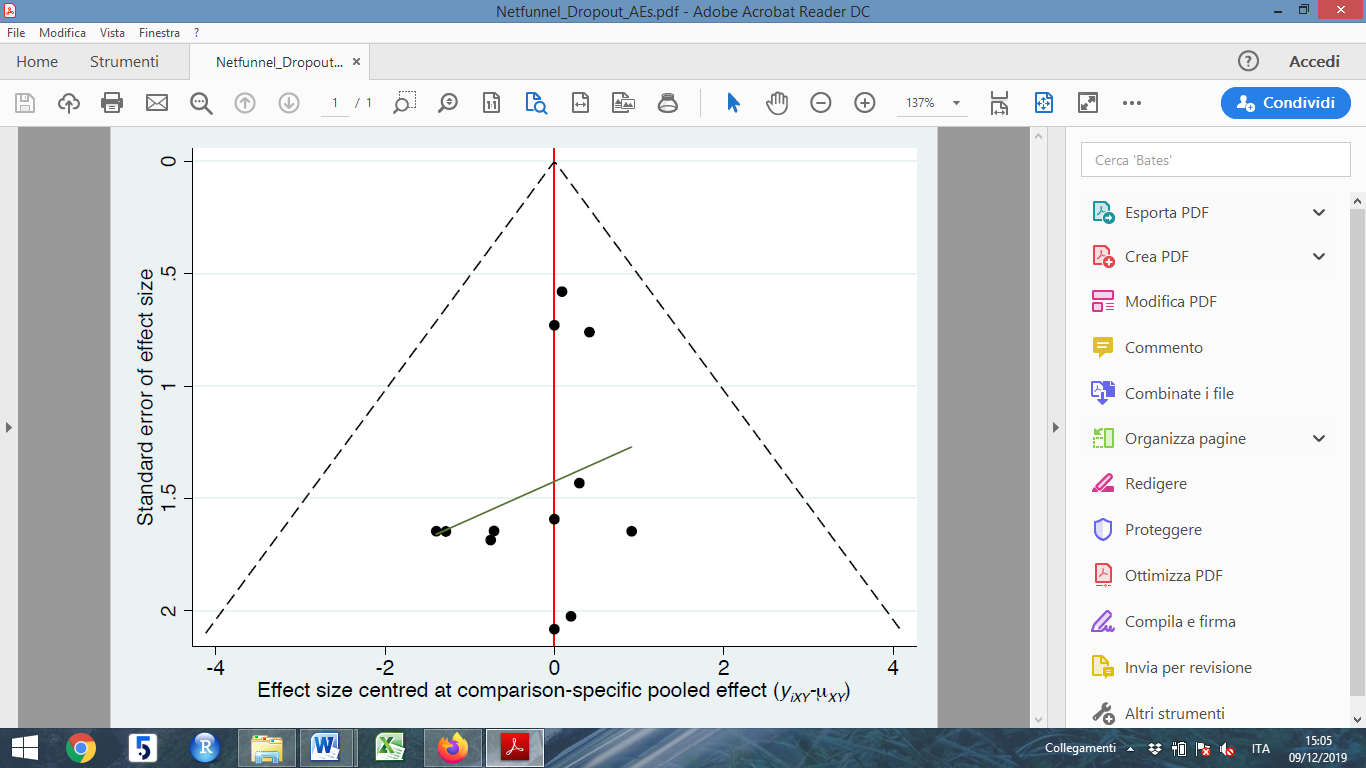

Supplement: Supplementary file 7 — Additional file 7: Funnel Plots for outcome with estimates for at least 8 studies. [file 12955_2021_1669_MOESM7_ESM.docx]
