# Supplementary material for: Impact of antipsychotics in children and adolescents with autism spectrum disorder: a systematic review and meta-analysis
Source: Health Qual Life Outcomes. 2021 Jan 25;19:33. doi: 10.1186/s12955-021-01669-0 (PMC7831175; doi:10.1186/s12955-021-01669-0)
Supplement: Supplementary file 8 — Additional file 8: GRADE Evidence profile. [file 12955_2021_1669_MOESM8_ESM.docx]

**Additional file 8: GRADE evidence profile**

**Question**: Should antipsychotics be used compared to not using antipsychotics for children and adolescents with ASD?

**Setting**: Inpatients and Outpatients

| **Certainty assessment** | | | | | | | **№ of patients** | | **Effect** | | **Certainty** | **Importance** |
| --- | --- | --- | --- | --- | --- | --- | --- | --- | --- | --- | --- | --- |
| **№ of studies** | **Study design** | **Risk of bias** | **Inconsistency** | **Indirectness** | **Imprecision** | **Other considerations** | **D2 blockers** | **no D2 blockers** | **Relative (95% CI)** | **Absolute (95% CI)** |  |  |
| **Restricted and repetitive interests and behaviors (follow up: median 8 weeks; assessed with: Aberrant Behaviour Checklist (ABC) - stereotypies; Children's Psychiatric Rating Scale (CPRS) (lower scores indicate improvement)** | | | | | | | | | | | | |
| 9 ^1,2,3,4,5,6,7,8,9^ | randomised trials | serious ^a^ | not serious | not serious | not serious | none | 492 | 331 | - | SMD **0.21 SD lower** (0.35 lower to 0.06 lower) | ⨁⨁⨁◯ MODERATE | CRITICAL |
| **Hyperactivity, inattention, oppositiveness, disruptive behavior (follow up: median 8 weeks; assessed with: Aberrant Behaviour Checklist (ABC)- hyperactivity (lower scores indicate improvement)** | | | | | | | | | | | | |
| 8 ^2,3,4,5,6,7,8,9^ | randomised trials | serious ^a^ | not serious | not serious | not serious | none | 473 | 310 | - | SMD **0.67 SD lower** (0.92 lower to 0.42 lower) | ⨁⨁⨁◯ MODERATE | CRITICAL |
| **Self-harm (follow up: mean 8 weeks; assessed with: Nisonger Child Behaviour Rating Form (N-CBRF)-Self-Injurial/stereotypic (lower scores indicate improvement)** | | | | | | | | | | | | |
| 1 ^8^ | randomised trials | serious ^b^ | not serious | not serious | very serious ^c^ | none | 39 | 38 | - | SMD **0.14 SD lower** (0.59 lower to 0.31 higher) | ⨁◯◯◯ VERY LOW | CRITICAL |
| **Social communication, social interaction (follow up: median 8 weeks; assessed with: Aberrant Behaviour Checklist (ABC) - social withdrawal, Childhood Autism Rating Scale (CARS), Discrimination Index (lower scores indicate improvement)** | | | | | | | | | | | | |
| 10 ^2,3,4,5,6,7,8,9,10,11^ | randomised trials | serious ^a^ | not serious | not serious | not serious | none | 506 | 348 | - | SMD **0.37 SD lower** (0.58 lower to 0.16 lower) | ⨁⨁⨁◯ MODERATE | CRITICAL |
| **Emotional dysregulation/irritability (follow up: median 8 weeks; assessed with: Aberrant Behaviour Checklist (ABC) -irritability (lower scores indicate improvement)** | | | | | | | | | | | | |
| 9 ^2,3,4,5,6,7,8,9,12^ | randomised trials | serious ^a^ | serious ^d^ | not serious | not serious | none | 534 | 345 | - | SMD **0.78 SD lower** (1.04 lower to 0.51 lower) | ⨁⨁◯◯ LOW | CRITICAL |
| **Anxiety (follow up: median 8 weeks; assessed with: Nisonger Child Behaviour Rating Form (N-CBRF) - Insecure/anxious (lower scores indicate improvement)** | | | | | | | | | | | | |
| 1 ^8^ | randomised trials | serious ^b^ | not serious | not serious | very serious ^c^ | none | 39 | 38 | - | SMD **0.38 SD lower** (0.83 lower to 0.07 higher) | ⨁◯◯◯ VERY LOW | CRITICAL |
| **Global functioning, global improvement (follow up: median 8 weeks; assessed with: Clinical Global Impression (CGI)- Severity, CGI-Improvement, Childhood Autism Rating Scale (CARS), Children's Global Assessment Scale (CGAS), Visual Analogue Scale (VAS) (lower scores indicate improvement)** | | | | | | | | | | | | |
| 10 ^2,3,4,7,8,11,12,13,14,15^ | randomised trials | serious ^e^ | serious ^f^ | not serious | not serious | none | 507 | 332 | - | SMD **0.64 SD lower** (0.96 lower to 0.33 lower) | ⨁⨁◯◯ LOW | CRITICAL |
| **Obsessions, compulsions (follow up: median 8 weeks; assessed with: Children Yale-Brown Obsessive Compulsive Scale (CY-BOCS) (lower scores indicate improvement)** | | | | | | | | | | | | |
| 4 ^2,3,4,7^ | randomised trials | not serious | not serious | not serious | serious ^g^ | none | 356 | 192 | - | SMD **0.3 SD lower** (0.55 lower to 0.06 lower) | ⨁⨁⨁◯ MODERATE | IMPORTANT |
| **Severe adverse events (follow up: median 8 weeks; assessed with: Number of patients with a serious/severe adverse event)** | | | | | | | | | | | | |
| 13 ^2,3,4,5,6,7,8,11,12,13,14,16,17^ | randomised trials | serious ^h^ | not serious | not serious | serious ^i^ | none | 13/623 (2.1%) | 7/434 (1.6%) | **RR 1.07** (0.47 to 2.41) | **1 more per 1.000** (from 9 fewer to 23 more) | ⨁⨁◯◯ LOW | CRITICAL |
| **Adverse events (follow up: median 8 weeks; assessed with: Number of patients with at least an adverse event)** | | | | | | | | | | | | |
| 10 ^1,2,3,4,7,8,12,13,16,17^ | randomised trials | serious ^j^ | not serious | not serious | not serious | none | 458/557 (82.2%) | 241/367 (65.7%) | **RR 1.19** (1.07 to 1.32) | **125 more per 1.000** (from 46 more to 210 more) | ⨁⨁⨁◯ MODERATE | CRITICAL |
| **Dropout due to any cause (follow up: median 8 weeks; assessed with: Number of patients who discontinued the treatment due to any cause)** | | | | | | | | | | | | |
| 15 ^1,2,3,4,5,6,12,13,14,16,17,18,19,20^ | randomised trials | serious ^k^ | not serious | not serious | not serious | none | 92/657 (14.0%) | 114/467 (24.4%) | **RR 0.61** (0.48 to 0.78) | **95 fewer per 1.000** (from 127 fewer to 54 fewer) | ⨁⨁⨁◯ MODERATE | IMPORTANT |
| **Dropout due to adverse events (follow up: median 8 weeks; assessed with: Number of patients who discontinued the treatment due to an adverse event)** | | | | | | | | | | | | |
| 12 ^2,3,4,5,12,13,14,16,17,19,20^ | randomised trials | serious ^l^ | not serious | not serious | serious ^i^ | none | 31/601 (5.2%) | 16/409 (3.9%) | **RR 0.99** (0.55 to 1.79) | **0 fewer per 1.000** (from 18 fewer to 31 more) | ⨁⨁◯◯ LOW | NOT IMPORTANT |

**CI:** Confidence interval; **SMD:** Standardised mean difference; **RR:** Risk ratio

#### Explanations

a. Downgraded by one level because most studies are at unclear risk of distortion for selection bias and one study is at serious risk of distortion for attrition bias

b. Downgraded by one level because the study is at unclear risk of distortion with respect to selection bias.

c. Downgraded by two levels because the study sample was small (<400) and there is a wide confidence interval that includes both appreciable benefits and noticeable damage

d. Downgraded by one level because for heterogeneity (I^2^= 71%), several confidence intervals of the included trial not overlapping and important differences in the point estimates.

e. Downgraded by one level because most of the studies are at unclear risk of distortion with respect to selection bias and one study is at serious risk of selection bias and two studies for reporting bias.

f. Downgraded by one level because for heterogeneity (I^2^= 75.6%)

g. Downgraded by one level because there is a wide confidence interval that includes both appreciable benefits and noticeable damage.

h. Downgraded by one level because most of the studies are at unclear risk of distortion with regard to selection bias and three studies are at serious risk of attrition bias, 1 at serious risk of selection bias and 1 at serious risk of reporting bias.

i. Downgraded by one level because there is a wide confidence interval that includes both appreciable benefits and noticeable damage.

j. Downgraded by one level because most studies are at unclear risk of distortion with respect to selection bias and two studies are at serious risk of attrition bias.

k. Downgraded by one level because most of the studies are at unclear risk of distortion with regard to selection bias and 4 studies are at serious risk of attrition bias, 1 at serious risk of selection bias and 1 at serious risk of reporting bias.

l. Downgraded by one level because most of the studies are at unclear risk of distortion with regard to selection bias, four studies are at serious risk of attrition bias, one at serious risk of selection bias and one at serious risk of reporting bias.

#### References

1. Campbell M, Anderson LT,Meier M,Cohen IL,Small AM,Samit C,Sachar EJ.. A comparison of haloperidol and behavior therapy and their interaction in autistic children. J Am Acad Child Psychiatry; 1978.

2. Ichikawa H, Mikami K,Okada T,Yamashita Y,Ishizaki Y,Tomoda A,et al.. Aripiprazole in the Treatment of Irritability in Children and Adolescents with Autism Spectrum Disorder in Japan: A Randomized, Double-blind, Placebo-controlled Study. Child Psychiatry Hum Dev; 2017.

3. Loebel A, Brams M,Goldman RS,Silva R,Hernandez D,Deng L,et al.. Lurasidone for the treatment of irritability with autistic disorder. J Autism Dev Disord; 2016.

4. Marcus RN, Owen R,Kamen L,Manos G,McQuade RD,Carson WH,Aman MG.. A placebo-controlled, fixed-dose study of aripiprazole in children and adolescents with irritability associated with autistic disorder. J Am Acad Child Adolesc Psychiatry; 2009.

5. McCracken JT, McGough J,Shah B,Cronin P,Hong D,Aman MG,et al.. Risperidone in children with autism and serious behavioral problems. N Engl J Med; 2002.

6. NCT00870727, Study of Aripiprazole in the Treatment of Pervasive Developmental Disorders. clinicaltrial.gov; 2009.

7. Owen R, Sikich L,Marcus RN,Corey-Lisle P,Manos G,McQuade RD,et al. Aripiprazole in the treatment of irritability in children and adolescents with autistic disorder. Pediatrics; 2009.

8. Shea S, Turgay A,Carroll A,Schulz M,Orlik H,Smith I,et al. Risperidone in the treatment of disruptive behavioral symptoms in children with autistic and other pervasive developmental disorders. Pediatrics; 2004.

9. Troost PW, Lahuis BE,Steenhuis MP,Ketelaars CE,Buitelaar JK,van Engeland H,et al. Long-term effects of risperidone in children with autism spectrum disorders: a placebo discontinuation study. J Am Acad Child Adolesc Psychiatry; 2005.

10. Anderson LT, Campbell M,Grega DM,Perry R,Small AM,Green WH.. Haloperidol in the treatment of infantile autism: effects on learning and behavioral symptoms. Am J Psychiatry; 1984.

11. Nagaraj R, Singhi P,Malhi P. Risperidone in children with autism: randomized, placebo-controlled, double-blind study. J Child Neurol; 2006.

12. Kent JM, Kushner S,Ning X,Karcher K,Ness S,Aman M,et al.. Risperidone dosing in children and adolescents with autistic disorder: a double-blind, placebo-controlled study. Journal of Autism and Developmental Disorders; 2013.

13. Luby J, Mrakotsky C,Stalets MM,Belden A,Heffelfinger A,Williams M,Spitznagel E,et al.. Risperidone in preschool children with autistic spectrum disorders: an investigation of safety and efficacy. J Child Adoles Psychopharmacol; 2006.

14. Hollander E, Wasserman S,Swanson EN,Chaplin W,Schapiro M.,Zagursky K,Novotny S.. A double-blind placebo-controlled pilot study of olanzapine in childhood/adolescent pervasive developmental disorder. J Child Adolesc Psychopharmacol; 2006.

15. Anderson LT, Campbell M,Adams P,Small AM,Perry R,Shell J.. The effects of haloperidol on discrimination learning and behavioral symptoms in autistic children. J Autism Dev Disord; 1989.

16. NCT01624675, . A Study to Evaluate the Efficacy and Safety of Risperidone (R064766) in Children and Adolescents With Irritability Associated With Autistic Disorder. clinicaltrial.gov; 2012.

17. Findling RL, Mankoski R,Timko K,Lears K,McCartney T,McQuade RD,et al. A randomized controlled trial investigating the safety and efficacy of aripiprazole in the long-term maintenance treatment of pediatric patients with irritability associated with autistic disorder. J Clin Psychiatry; 2014.

18. Nagaraj R, Singhi P,Malhi P.. Risperidone in children with autism: randomized, placebo-controlled, double-blind study. J Child Neurol; 2006.

19. Shea S, Turgay A,Carroll A,Schulz M,Orlik H,Smith I,Dunbar F.. Risperidone in the treatment of disruptive behavioral symptoms in children with autistic and other pervasive developmental disorders. Pediatrics; 2004.

20. Remington G, Sloman L,Konstantareas M,Parker M,Gow R.. Clomipramine versus haloperidol in the treatment of autistic disorder: a double-blind, placebo-controlled, crossover study. J Clin Psychopharmacol; 2001.
